# Rapid Quantification of *C. difficile* Glutamate Dehydrogenase and Toxin B (TcdB) with a NanoBiT Split-Luciferase Assay

**DOI:** 10.1021/acs.analchem.1c05206

**Published:** 2022-05-28

**Authors:** Hope Adamson, Modupe O. Ajayi, Kate E. Gilroy, Michael J. McPherson, Darren C. Tomlinson, Lars J. C. Jeuken

**Affiliations:** † School of Biomedical Sciences and Astbury Centre for Structural Molecular Biology, University of Leeds, Leeds, LS2 9JT, United Kingdom; ‡ School of Molecular and Cellular Biology and Astbury Centre for Structural Molecular Biology, University of Leeds, Leeds, LS2 9JT, United Kingdom; § Leiden Institute of Chemistry, Leiden University, PC Box 9502, 2300 RA, Leiden, The Netherlands

## Abstract

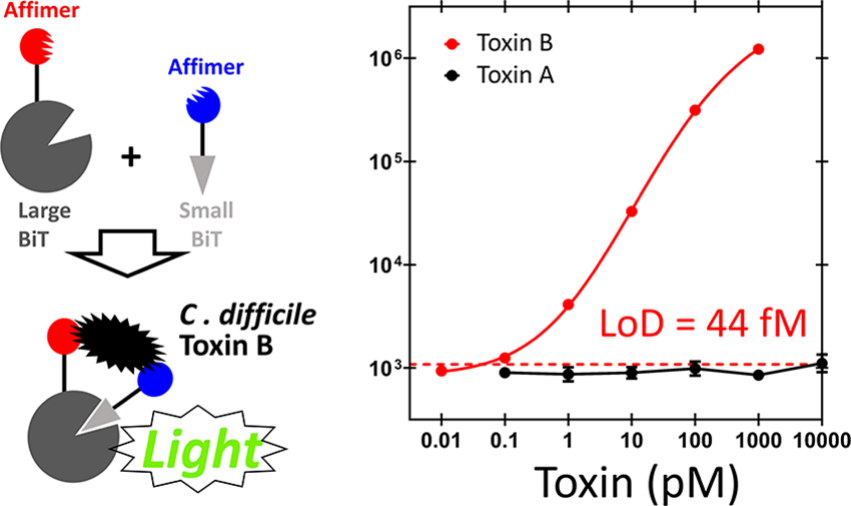

*C. difficile* infection (CDI) is a leading healthcare-associated infection with a high morbidity and mortality and is a financial burden. No current standalone point-of-care test (POCT) is sufficient for the identification of true CDI over a disease-free carriage of *C. difficile*, so one is urgently required to ensure timely, appropriate treatment. Here, two types of binding proteins, Affimers and nanobodies, targeting two *C. difficile* biomarkers, glutamate dehydrogenase (GDH) and toxin B (TcdB), are combined in NanoBiT (NanoLuc Binary Technology) split-luciferase assays. The assays were optimized and their performance controlling parameters were examined. The 44 fM limit of detection (LoD), 4–5 log range and 1300-fold signal gain of the TcdB assay in buffer is the best observed for a NanoBiT assay to date. In the stool sample matrix, the GDH and TcdB assay sensitivity (LoD = 4.5 and 2 pM, respectively) and time to result (32 min) are similar to a current, commercial lateral flow POCT, but the NanoBit assay has no wash steps, detects clinically relevant TcdB over TcdA, and is quantitative. Development of the assay into a POCT may drive sensitivity further and offer an urgently needed ultrasensitive TcdB test for the rapid diagnosis of true CDI. The NanoBiTBiP (NanoBiT with Binding Proteins) system offers advantages over NanoBiT assays with antibodies as binding elements in terms of ease of production and assay performance. We expect this methodology and approach to be generally applicable to other biomarkers.

## Introduction

*Clostridioides* (formerly *Clostridium*) *difficile* is an anaerobic, Gram-positive bacillus that is a leading cause of healthcare-associated infections with high morbidity and high mortality.^1^ Transmission is via spores by the fecal-oral route and disruption of protective intestinal microbiota by prior antibiotic administration is a major risk factor for *C. difficile* infection (CDI).^2,3^ The main virulence factors are toxin A (TcdA) and toxin B (TcdB), which trigger a cascade of host cellular responses that can lead to significant intestinal damage.^4^ Symptoms range from mild, self-limiting diarrhea to severe, life-threatening colitis.^1^ In the U.S. alone, the annual burden is estimated to be over 600000 episodes, 44500 deaths, and $5.4 billion in costs, largely due to hospitalization.^5^ The severity and frequency of CDI has increased over the last two decades, and methods to reduce this burden are urgently required.^1,6^

The timely and accurate diagnosis of CDI is imperative to ensure effective treatment and implementation of infection control measures. The disease-free carriage of *C. difficile* is widespread, and it is important to distinguish this from true CDI.^7−9^ Current clinical guidance is to first use a high sensitivity stool test (e.g., an enzyme immunoassay (EIA) for common *C. difficile* antigen glutamate dehydrogenase (GDH)), for which a negative result reliably rules out CDI.^8^ Positives are followed up with a high specificity stool test (e.g., EIA for disease causing toxins TcdA/TcdB), for which a positive result reliably confirms CDI.^8^ GDH and toxin immunoassays have been combined in lateral-flow tests (LFTs, e.g., *C. diff.* Quik Chek Complete), offering a simple, rapid, point-of-care approach to CDI diagnosis.^10^ However, the toxin test has low sensitivity, and GDH+/toxin− results can be due to CDI with low toxin levels or *C. difficile* carriage, so further clinical evaluation and testing are required.^8,10^ Patients with CDI may suffer life-threatening delays in treatment and infection control, while carriers may be inappropriately prescribed antibiotics that in fact make them more vulnerable to CDI and drug-resistant pathogens.^11^ Thus, a more sensitive rapid toxin test would vastly improve CDI diagnosis and improve patient outcomes. Quantitative tests, rather than qualitative LFTs, may also prove useful for research into correlates with disease outcomes and optimal treatment.

**Figure 1:**
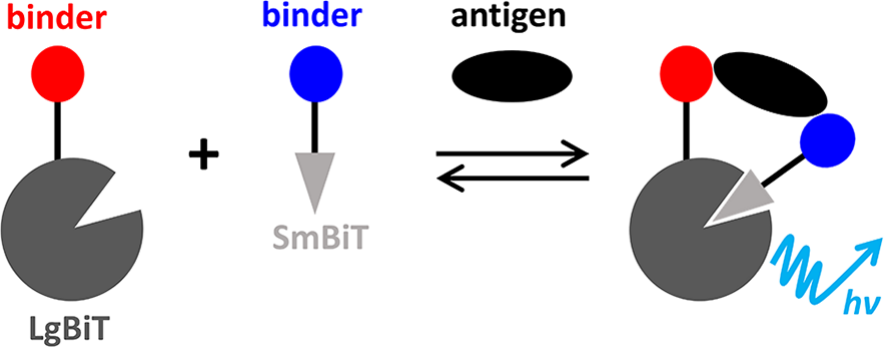
Schematic of the NanoBiT split-luciferase assay. Fragments of the split NanoLuc enzyme, LgBiT and SmBiT, are attached to binding proteins that target different regions of the analyte. Analyte binding colocalizes LgBiT and SmBiT, promoting reconstitution of the enzyme and bioluminescence upon addition of Nano-Glo substrate.

A promising technology for sensitive and quantitative point-of-care tests (POCTs) is the NanoBiT split-luciferase assay. Binding elements are tethered to small (SmBiT) and large (LgBiT) fragments of the engineered luciferase NanoLuc, such that analyte binding induces fragment colocalization and reconstitution of active enzyme (Figure 1).^12−14^ This rapid, homogeneous, wash-free assay has a simple mix-and-read format, and the bioluminescent output can even be read with a camera, so it is well suited to adoption in POCTs.^14^ The NanoBiT fragments have been engineered for weak background affinity and high reconstituted bioluminescent activity, maximizing assay sensitivity and dynamic range.^12−14^ Recent NanoBiT immunoassays with approximately 30 min time scales have reliably quantified several protein biomarkers and antibodies over concentrations spanning 4 orders of magnitude, with low pM sensitivity and up to a 1000-fold signal to background ratios.^14,15^ Analytical performance approaches or exceeds laboratory EIAs, but with the ease and speed of LFTs.^14,15^

Here, we generate NanoBiT assays for *C. difficile* biomarkers GDH and TcdB. We do not target TcdA, as TcdB is able to independently cause disease, and many clinical isolates are TcdA–/TcdB+, but not TcdA+/TcdB–.^16^ NanoBiT immunoassays targeting protein biomarkers utilize antibodies as the binding element.^13,14^ This is convenient, as they are commercially available, but necessitate chemical conjugation to each NanoBiT fragment, which can complicate manufacturing.^13,14^ In this work, we have thus tested nanobodies^17^ (single domain antibodies) and Affimers^18^ (synthetic nonimmunoglobulin binding proteins) as attractive alternatives, as they are small, stable, and easily recombinantly produced in fusion with protein sensors like NanoBiT fragments.^19,20^ They have been selected against a broad range of analytes with high affinity and specificity by in vitro display methods, so development for new targets is straightforward.^17,18^ Their small size relative to antibodies may also allow colocalization of NanoBiT fragments in a smaller volume, improving reconstitution and assay performance. We term these assays NanoBiTBiP (NanoBiT with Binding Proteins) and explore performance controlling parameters for optimization. Rapid tests developed for *C. difficile* GDH and TcdB offer analyte quantification over concentrations spanning 4–5 orders of magnitude, with low pM–fM sensitivity, up to 1300-fold signal to background ratios, and compatibility, though limited, with stool samples. Development into a POCT may offer an urgently required improvement in *C. difficile* diagnostic and research tools. We expect NanoBiTBiP to be widely applicable to further biomarkers and offer a promising underpinning assay technology for POCTs.

## Experimental Section

Affimer selection, validation, characterization by SPR, and generation of sensor constructs by standard restriction cloning methods are described in detail in the Supporting Information.

### Sensor Expression and Purification

The pET28a vectors with sensor constructs containing Affimers were transformed into *E. coli* BL21* (DE3) cells, and those containing nanobodies were transformed into *E. coli* SHuffle T7 cells (NEB). A 1 mL starter culture was added to 50 mL of LB media (with 50 μg mL^–1^ kanamycin) and grown at 37 °C, 220 rpm before induction at OD600 of about 0.6 with 0.3 mM isopropyl-β-d-thiogalactoside (IPTG) and overnight growth at 16 °C, 180 rpm. Cells were harvested at about 4000 g for ∼20 min, resuspended in 4 mL of lysis buffer (pH 7.4, 50 mM Tris, 300 mM NaCl, 10 mM imidazole, 0.1 mg mL^–1^ lysozyme, 1× cOmplete EDTA-free protease inhibitor (Merck), 0.001% v/v benzonase nuclease (Merck)), and incubated on a roller mixer for 1 h at 4 °C. Cells were lysed by sonication (UP50H, Hielscher) for 2 min (5 s on/5 s off) at 100% amplitude and then pelleted at about 17000 g for 20 min. The supernatant was added to 250 μL of Super Co-NTA resin (Generon) that had been pre-equilibrated with wash buffer (pH 7.4, 50 mM Tris, 300 mM NaCl, 10 mM imidazole) and was then incubated on a roller mixer for 1 h at 4 °C. The resin was washed thrice with 5 mL of wash buffer and protein eluted with 3 × 0.5 mL of elution buffer (pH 7.4, 50 mM Tris, 300 mM NaCl, 300 mM imidazole). Pure fractions (as assessed by SDS-PAGE) were buffer exchanged into storage buffer (50 mM Tris, 150 mM NaCl, pH 7.4) using Zeba spin desalting columns (ThermoFisher). Protein concentration was determined by a BCA assay, and the aliquots were stored at −80 °C.

### Sensor Characterization

#### NanoBiT Assay (in Buffer)

All assays were performed in PBSB (pH 7.4, PBS + 1 mg mL^–1^ BSA) dilution buffer. A total of 10 μL of LgBiT sensor (5× final conc.), 10 μL of SmBiT sensor (5× final conc.), and 5 μL of TxB or GDH (10× final conc.) were added to a well of a white, no-bind, 384-well plate (Corning) and incubated, shaking at 25 °C, for the indicated length of time. Then, 25 μL of diluted Nano-Glo (2× final conc.) was added, and the luminescence was read (500 ms integration) on a Tecan Spark plate reader. Data were fit to five parameter logistic (5PL) regression curves, and interpolations were made using GraphPad Prism 9 software.

#### NanoBiT Assay (in Fecal Sample Matrix)

*C. difficile* negative fecal samples were excess routinely collected diagnostic specimens from the Department of Microbiology, Leeds Teaching Hospitals NHS Trust. Samples were anonymized by the clinical team prior to the storage of two 1 mL aliquots at −80 °C, until transfer was made to the research team for testing (REC reference 17/LO/2099).

All sample preparation and assays were performed in PBSBT (pH 7.4, PBS + 1 mg mL^–1^ BSA + 0.05% Tween), unless otherwise stated. Fecal samples of 125 mg were homogenized in 750 μL of buffer (16.67% w/v). Particulates were pelleted by centrifugation at about 17000 *g* for 5 min (or allowed to settle for 10 min, if stated), and the supernatant was used as the fecal sample, which was added to the NanoBiT assay to give the indicated final concentrations (w/v). Typically, a 1:5 dilution was used to give 3.33% (w/v) feces, as follows: 10 μL of LgBiT + SmBiT sensor mix (5x final conc.), 5 μL of TxB or GDH (10× final conc.), and 10 μL of fecal sample were added to a well of a white, no-bind, 384-well plate (Corning) and incubated, shaking at 25 °C, for 30 min (or the indicated length of time). Then 25 μL of diluted Nano-Glo (2× final conc.) was added, and the luminescence was read (500 ms integration) on a Tecan Spark plate reader. We note that a final stool concentration of 3.33% (w/v) is equivalent to that used in the commercial *C. diff.* Quik Chek complete test (Alere), which was used in this study as a comparison. All data refer to the final concentration of analyte present in the final assay mixture.

The *C. diff.* Quik Chek complete test (Alere) was performed according to the manufacturer’s instructions.

## Results and Discussion

### Selection and Characterization of Binding Proteins

For both GDH and TcdB, a pair of binding proteins targeting distinct regions of the biomarker are required for use in the split-luciferase assay (Figure 1). A literature search identified two nanobodies, E3 and 7F, which bind different regions of TcdB.^21^ We isolated Affimer binding proteins targeting GDH and TcdB to complement the nanobody binders. An Affimer phage display library^22^ was screened against biotinylated GDH or TcdB with three rounds of panning. To improve the specificity of Affimers, phages for the GDH screen were prepanned against cell lysate, and the third panning round for TcdB included a competitive incubation with TcdA to remove cross-reactive binders. After the third pan, individual clones were screened by phage ELISA, and hits were classified as wells with a more than 2-fold increase in signal relative to controls (TcdA for TcdB and cell lysate for GDH). All hits were sequenced, and unique Affimer reagents were produced and purified. Binding to TcdB or GDH was assessed by ELISA (Figures S2A and S3A). Affimers that showed the highest signal by ELISA were taken forward, and pairwise binding of other Affimers was assessed by sandwich ELISA (Figures S2B and S3B). Affimers 18 and 45 were identified as the best pair to bind distinct sites of TcdB. No binding was observed in negative controls or against TcdA. GDH is hexameric, and Affimer 4 was demonstrated as the best capture and detection reagent.

Kinetic and equilibrium affinity constants of the binding proteins were determined by surface plasmon resonance (SPR; Figure S4 and Table 1). A biotinylated Affimer/nanobody was immobilized on a streptavidin chip, titrated with serial dilutions of analyte, and the association/dissociation response was fit to 1:1 Langmuir model. All binding proteins were specific for their target analyte (Figure S4) and displayed nM binding affinity (Table 1). Affimer selection and validation was with native toxin B (TcdB), while SPR and further sensor characterization was with commercially available inactivated toxoid B (TxB) that maintains antigenicity.

**Table 1:** Kinetic and Equilibrium Affinity Binding Constants of Affimers and Nanobodies Derived from SPR Data^a^

binding protein	target	*k*_a_ ± SE	*k*_d_ ± SE	*K*_d_ ± SE
		(M^–1^ s^–1^) ×10^4^	(s^–1^) ×10^–4^	(nM)
Affimer 4	GDH	4.4 ± 0.8	8.1 ± 0.1	19 ± 3.2
Affimer 18	TxB	14 ± 3	38 ± 11	26 ± 2.6
Affimer 45	TxB	7.7 ± 1.2	9.4 ± 0.3	13 ± 2.4
Nanobody E3	TxB	10 ± 1	2.5 ± 0.2	2.5 ± 0.04
Nanobody 7F	TxB	2.8 ± 0.5	9.0 ± 0.1	33 ± 6.1

a Data are from duplicate (Affimer 4, Affimer 45, and nanobody 7F) or triplicate (nanobody E3) analyte titrations on one chip. Data for Affimer 18 are from three analyte titrations across two chips. Standard errors from the mean are shown.

### Development of Split-Luciferase Assays

The NanoBiT system consists of an 18 kDa LgBiT and a range of 11–13 amino acid SmBiT peptides that span a 5 order of magnitude binding affinity for LgBiT.^12^ Here we use SmBiT101 (VTGYRLFEKES) with *K*_d_ = 2.5 μM, to provide a balance between minimizing the background complementation and maximizing analyte-induced reconstitution.^12,14^ To generate NanoBiT sensor proteins, each of the binding proteins (TcdB Affimers 18 and 45, TcdB nanobodies E3 and 7F, and GDH Affimer 4) were genetically fused to the N- or C-terminus of LgBiT (L) or SmBiT101 (S) via a (GSG)_7_ linker peptide. (Hereafter, we will use L-45 and S-45 for an N-terminal fusion of LgBiT and SmBiT to Affimer 45, respectively, and similarly, E3-L and E3-S were used for a C-terminal fusion to nanobody E3 and so on. The linker, (GSG)_7_, is present in all constructs but not included in this nomenclature.) Of these 20 constructs, 16 were successfully produced in *E. coli* and purified via a C-terminal 6x-Histag (Figure S5) with yields of up to 90 mg L^–1^, demonstrating the ease of production relative to antibody-based systems. All four constructs with N-terminal nanobodies (E3-L, E3-S, 7F-L, and 7F-S) were produced with insufficient purity following metal affinity purification (Figure S5), so were not taken forward for use in assays.

**Figure 2:**
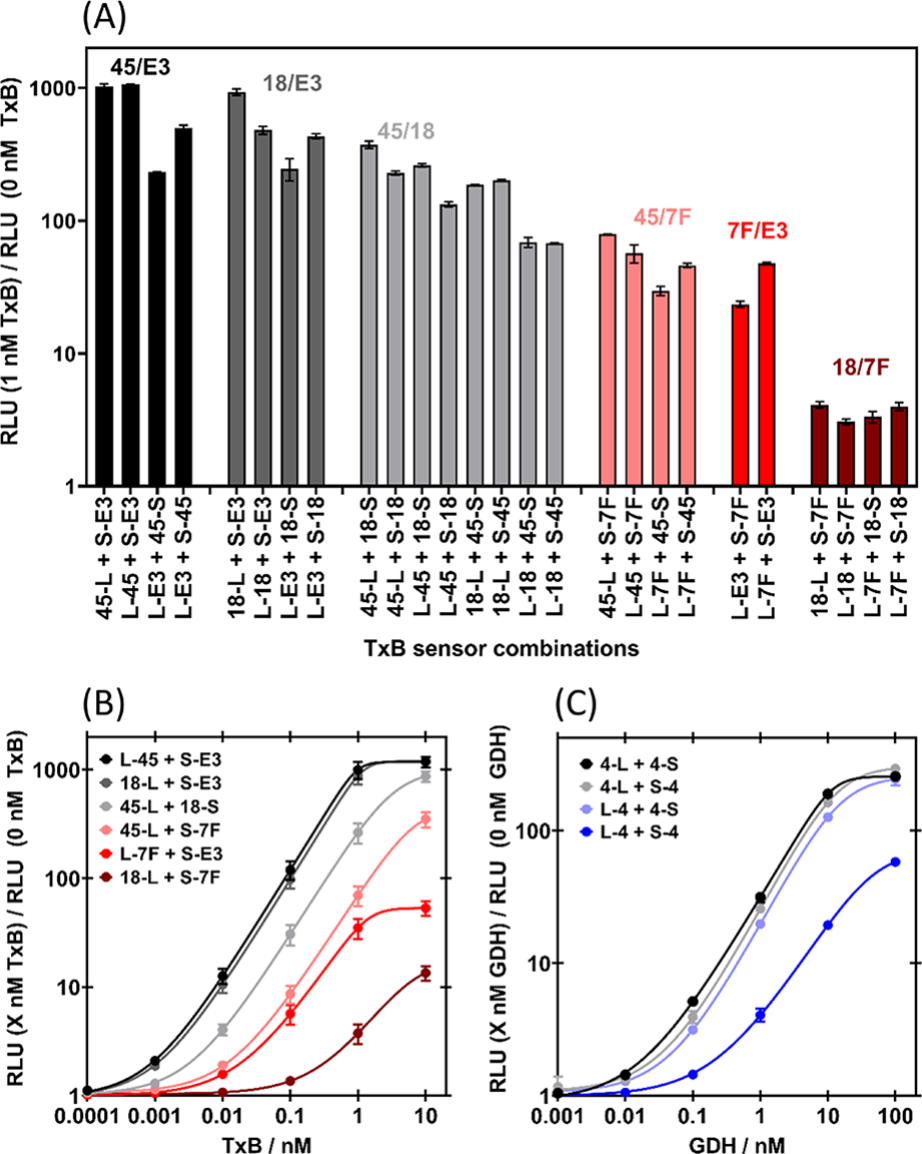
Establishing optimal sensor protein combinations. (A) Fold gain in bioluminescence of TxB sensor proteins with 1 nM TxB vs 0 nM TxB. Luminescence was read immediately after substrate addition and data are the mean of duplicates on the same plate. Different colors indicate different binding protein pairs. (B) Dose response of TxB sensor proteins. Luminescence was read 4 min after substrate addition and data are the mean of three independent measurements. (C) Dose response of GDH sensor proteins. Luminescence was read 4 min after substrate addition and data are the mean of two sets of duplicates from two independent experiments. For all assays, analyte (final concentration indicated) and sensor proteins (final concentration = 2 nM each) were incubated for 30 min, at 25 °C, with agitation prior to addition of Nano-Glo substrate to a final dilution of 1:1000. Error bars indicate standard deviation from the mean and solid lines are 5PL regression fits (B: 0.970 < *R*^2^ < 0.987 and C: 0.990 < *R*^2^ < 0.999).

To assess which combination of LgBiT and SmBiT sensor proteins have optimal TxB driven complementation, each pair was incubated with 0 or 1 nM TxB prior to the addition of the Nano-Glo substrate and measurement of bioluminescence. The increase in bioluminescence with 1 nM TxB ranged from only 4-fold for sensor pairs containing Affimer 18 and nanobody 7F up to over 1000-fold for some containing Affimer 45 and nanobody E3 (Figure 2A). Fusion of binding proteins at the N- or C-terminus of either LgBiT (L) or SmBiT (S) affected the TxB driven signal increase. For example, L-45 + S-E3 and L-E3 + 45-S displayed 1000- and 230-fold increases, respectively, despite containing the same binding proteins. Sensors were also assayed for bioluminescence across a wide range of TxB concentrations to give dose–response curves (Figure 2B). Thermodynamic modeling (Figure S6) indicates that, for binding protein with *K*_d_ values between 2.5 and 33 nM (Table 1), the expected sensitivities are almost identical and differences in *K*_d_ do not explain the differences in sensitivity observed in Figure 2B. Instead, the molecular mechanism behind the differences in signal may be due to some sensor pairs orienting more favorably for complementation of LgBiT and SmBiT. Alternatively, the engineering of LgBiT and SmBiT on the N- or C-terminus could alter the binding affinity of the Affimers or nanobodies.

Sensors L-45 + S-E3 displayed the highest TxB driven signal increase across the entire concentration range, so were taken forward for further optimization. All GDH sensor combinations displayed GDH-dependent bioluminescence (Figure 2C). The response was again dependent on Affimer placement at the N- or C-terminus of sensors, highlighting the importance of testing all combinations. Sensors 4-L + 4-S were optimal, so they were taken forward for further improvement.

**Figure 3:**
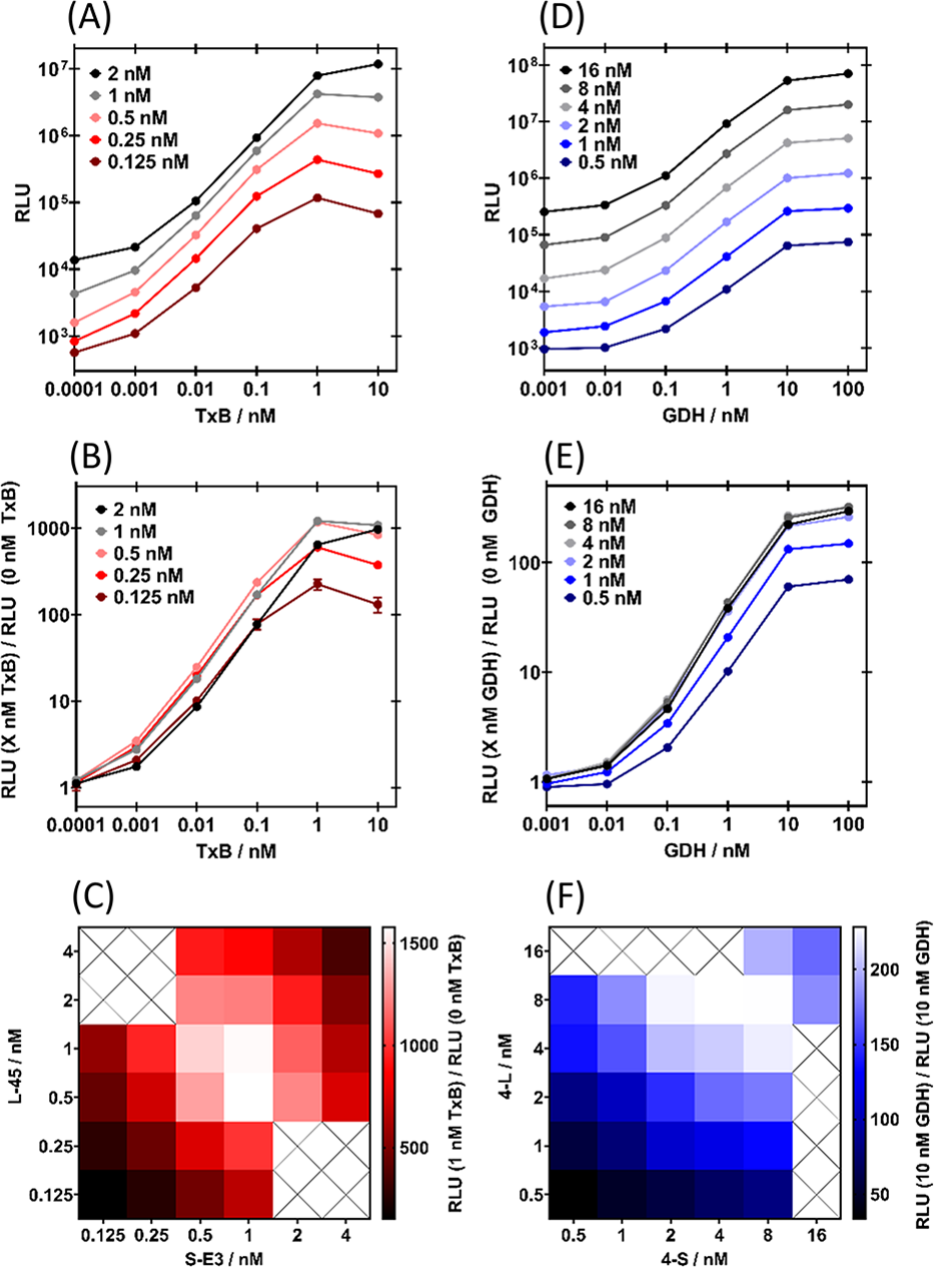
Establishing optimal sensor protein concentrations. (A) Bioluminescence and (B) fold gain in bioluminescence of 0.125–2 nM each of S-E3 + L-45 with TxB. (C) Heat map of fold gain in bioluminescence of 0.125–4 nM S-E3 + 0.125–4 nM L-45 with 1 nM TxB. (D) Bioluminescence and (E) fold gain in bioluminescence of 0.5–16 nM each of 4-S + 4-L with GDH. (F) Heat map of fold gain in bioluminescence of 0.5–16 nM 4-S + 0.5–16 nM 4-L with 10 nM GDH. For all assays, analyte (final concentration indicated) and sensor proteins (final concentration indicated) were incubated for 30 min, at 25 °C, with agitation prior to the addition of the Nano-Glo substrate to a final dilution of 1:1000, and bioluminescence was immediately read. For (A) and (B), data are the mean of triplicates on the same plate, and error bars (which for most data lies within the point) indicate a standard deviation from the mean. For (D) and (E), data are single measurements. For (C) and (F), data are the mean of duplicates on the same plate.

The optimal concentrations of sensor proteins for TxB (L-45 + S-E3) and GDH (4-L + 4-S) were then established (Figures 3 and S7). Lower concentrations of LgBiT and SmBiT minimize background complementation (Figure 3A,D) but reduce the maximum amount of analyte driven reconstitution (Figure 3A,D). There is also a more pronounced “Hook” effect (loss in signal at high analyte concentrations) due to analyte binding each sensor protein individually rather than in a sandwich complex. Therefore, there is an optimal sensor concentration that maximizes the ratio of analyte-induced to background bioluminescence (Figure 3B,E). Unequal concentrations of LgBiT and SmBiT were also tested (Figures 3C,F and S7A). For TxB, 0.5 nM S-E3 + 1 nM L-45 was established as the best combination, as it is the most sensitive at low TxB concentrations (Figure S7A). A combination of 8 nM 4-S + 8 nM 4-L performed the best for GDH (Figure 3E,F).

The kinetics of the NanoBiT assays were then studied (Figures S8 and S9) to optimize incubation and signal read times. When TxB sensors (S-E3 + L-45) were simultaneously mixed with TxB and substrate, the bioluminescent signal observed with 1–1000 pM TxB increased over a period of 30 min (Figure S8B,E), indicating the time scale to approach equilibrium. A 30 min preincubation with TxB prior to substrate addition gave an optimal bioluminescent signal (Figure S8A,D) that was stable over 30 min (Figure S8C,F). A stable signal is possible due to the glow-type luminescence of NanoLuc^23^ and makes the assay results less sensitive to the exact measurement time adopted by the user. Only a 15 min preincubation of GDH sensors (4-S and 4-L) with GDH was required to maximize the bioluminescent signal (Figure S9A,C), indicating slightly faster kinetics. The signal was, however, less stable over time (Figure S9B,D), and a read time of 0–2 min was optimal. We adopted a preincubation time of 30 min and a read time of 2 min for a consistent protocol between TxB and GDH assays. It should be noted that the preincubation can be removed to give a simple mix-and-read protocol if needed for a POCT, but the signal will just require time to develop.

Finally, the concentration of substrate was optimized by performing the TxB assay with 1:100–1:4000 Nano-Glo (Figure S10). Lower substrate concentrations minimized background luminescence (Figure S10A), so they maximized the fold gain in bioluminescence with TxB (Figure S10C). However, the signal also decreased more quickly over time with low Nano-Glo concentrations (Figure S10B,D), presumably due to a depletion of the substrate. A substrate concentration of 1:1000 balanced maximizing the signal increase with TxB and minimizing signal loss over time.

### Quantification of *C. difficile* Toxin B and GDH

**Figure 4:**
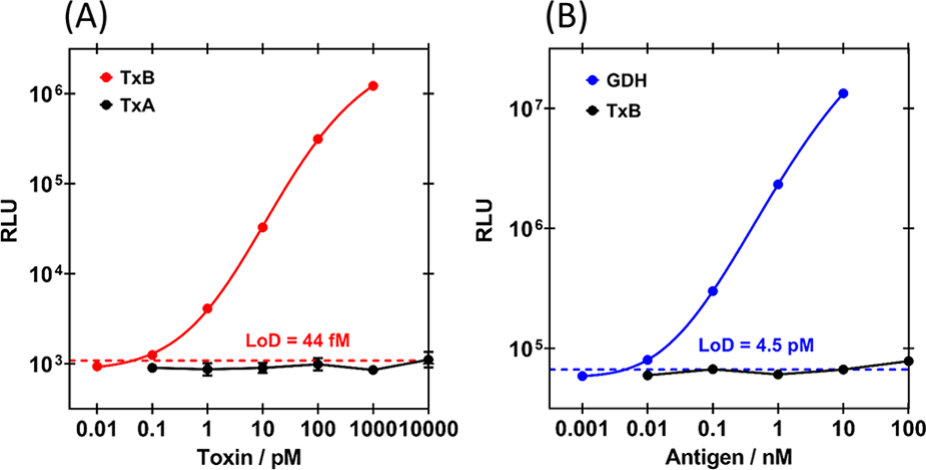
Optimized dose–response curves used to calculate intra-assay LoD, accuracy (% recovery), and precision (% CV). (A) Bioluminescent response of TxB split-luciferase assay with 0.5 nM S-E3 + 1 nM L-45. Data are the mean of six (TxB, red) or two (TxA control, black) replicates on the same plate. (B) Bioluminescent response of GDH split-luciferase assay with 8 nM 4-S + 8 nM 4-L. Data are the mean of six (GDH, blue) replicates on the same plate or single measurements (TxB control, black). For all assays, analyte (final concentration indicated) and sensor proteins (final concentration indicated) were incubated for 30 min at 25 °C, with agitation prior to the addition of the Nano-Glo substrate, to a final dilution of 1:1000, and bioluminescence was read after 2 min. Error bars (which for most data lie within the point) indicate the standard deviation from the mean, solid lines are 5PL regression fits (*R*^2^ = 1.000 for both A and B), and LoD are indicated by the dashed line.

Each assay requires a target specific LgBiT and SmBiT sensor protein (Figure S11A,B), confirming the expected need to form a sandwich complex with the analyte for luciferase reconstitution. The optimized split-luciferase assays for *C. difficile* TxB and GDH are specific for their target analytes, with no nonspecific response with up to 10× the concentration of TxA and TxB, respectively (Figure 4). Many commercial assays detect toxin but do not differentiate TcdA and TcdB, so this assay offers an advantage in specifically quantifying the more clinically relevant TcdB.^16^

The TxB and GDH assays were performed with nominal concentrations of TxB (0.01–1000 pM) and GDH (0.001–10 nM) to establish dose–response curves and calculate sensitivity, accuracy, and precision metrics (Figures 4 and S11). Responses were recorded as raw bioluminescence (RLU; Figures 4 and S11C,D) or fold gain in bioluminescence (RLU with analyte/RLU without analyte; Figure S11A,B,E,F). There were approximately 250- and 1300-fold maximum signal gains for the GDH and TxB assays, respectively. To calculate intra-assay metrics, six replicates were performed on the same plate (Figures 4S11A,B), and for interassay metrics, six independent measurements were made for TxB and three were made for GDH (Figure S11C–F). The logarithm of each dose response was fit to a five parameter logistic (5PL) regression standard curve. The limit of detection (LoD) was calculated by LoD = mean_blank_ + 1.645 (SD_blank_) + 1.645 (SD_low conc._), as outlined by Armbruster and Pry,^24^ where blank = zero analyte and low conc. = 0.1 pM TxB or 0.01 nM GDH, to account for variability in both test and blank measurements. For each individual measurement at nominal concentrations, the concentration was interpolated back from the standard curve to assess accuracy (% recovery = (mean interpolated concentration/nominal concentration) × 100%) and precision (% coefficient of variation (% CV) = (SD interpolated concentration/mean interpolated concentration) × 100%).

Intra-assay sensitivity, accuracy, and precision metrics were optimal with raw bioluminescence rather than fold gain data (Table S1), perhaps due to variability in low background luminescence. The intra-assay LoD was 44 fM for TxB and 4.5 pM for GDH (Figure 4, Table 2). Intra-assay recovery and CV values indicate good intra-assay accuracy and precision for analyte quantification over concentrations spanning 5 orders of magnitude for TxB (0.1–1000 pM) and 4 for GDH (0.01–10 nM). The interassay LoD was 190 fM for TxB and 14 pM for GDH (Figure S11C,D, Table 2). Good interassay accuracy and precision metrics are maintained over 3 orders of magnitude (1–100 pM for TxB and 0.1–10 nM for GDH, Table 2). Interassay sensitivity, accuracy, and precision metrics were further improved when using fold gain rather than raw bioluminescence data (Figure S11E,F, Table S1), as the zero analyte measurement acts as a calibrator for condition changes between assays.

**Table 2:** Sensitivity (LoD), Accuracy (% Recovery), and Precision (% CV) of TxB and GDH Assays, as Determined from Raw Bioluminescence (RLU; Figures 4 and S11C,D)

	intra-assay	interassay
TxB		
sensitivity (LoD)	44 fM	190 fM
quantifiable range	0.1–1000 pM	1–100 pM
% recovery	88–105%	99–101%
% CV	3–25%^a^	11–20%
GDH		
sensitivity (LoD)	4.5 pM	14 pM
quantifiable range	0.01–10 nM	0.1–10 nM
% recovery	100%	101–102%
% CV	1–10%	19–24%^a^

a %CV precision metrics > 20% only at the limit of quantification.

NanoBiT immunoassays have previously shown much improved signal gain, sensitivity, and range compared to other protein switch sensors, because the NanoBiT fragments have been extensively engineered for high reconstituted bioluminescent activity and weak background affinity.^12−15,20^ The 1300-fold signal gain and 44 fM LoD for TxB are the best metrics observed for a NanoBiT immunoassay to date. This is likely due to the high affinity of the Affimer and nanobody binders, while their small size may reduce the volume in which NanoBiT fragments colocalize, enhancing reconstitution relative to antibody-based systems. Thermodynamic analysis (Supporting Information, Modeling) suggested that engineering SmBiT and LgBiT onto the Affimers and nanobodies may influence either the reconstitution of functional NanoBiT or, alternatively, strongly affect the binding affinity of the Affimers/nanobodies. Without structural data, it is difficult to rationally design the best sensor constructs, and some trial-and-error optimization might be required for the best sensitivity. Further improvements in assay performance may be achieved with a recently developed ternary split-NanoLuc, consisting of a 17 kDa fragment and two peptides fused to binding elements.^25,26^ Background activity is further reduced, enhancing sensitivity, and the components can be lyophilized into a convenient, shelf-stable, add-and-read reagent.^25^ We have also highlighted that interassay accuracy and precision metrics can be brought into acceptable ranges with fold-gain measurements that use a single zero-analyte calibrator well and predetermined standard curve rather than a full calibration curve being required for each assay. A more convenient approach may be to include a recently developed internal calibrator, consisting of NanoLuc fused to a green fluorescent protein.^14^ The ratio of blue (NanoBiT) to green (calibrator) light is then consistent over time and conditions.^14^

### Assay Performance in a Stool Sample Matrix

TxB and GDH are present in stool samples for patients with CDI, so the effect of this sample matrix on the split-luciferase assay was assessed. We homogenized a solid *C. difficile* negative stool in buffer to 16.66% w/v and added this matrix to the assay at the final % w/v indicated, along with analyte to the final concentration indicated. A currently used *C. difficile* POCT, *C. diff.* Quik Chek complete (Quik Chek, Alere) uses a similar procedure to homogenize stool samples at 3.33% w/v.^27^

The TxB assay was initially performed with 0.66% w/v stool in a number of different buffers and PBSBT (PBS + 1 mg mL^–1^ + 0.05% Tween 20) was found to be optimal (Figure S12A). Pelleting stool particulates with centrifugation rather than allowing them to settle by gravity prior to addition to the assay minimized the signal loss by scattering and absorption of light (Figure S12B). Nano-Glo substrate at 1:1000 (Figure S12B) and approximately 1 nM TxB sensor proteins (Figure S13) were still optimal, with a 0.66% w/v stool matrix.

**Figure 5:**
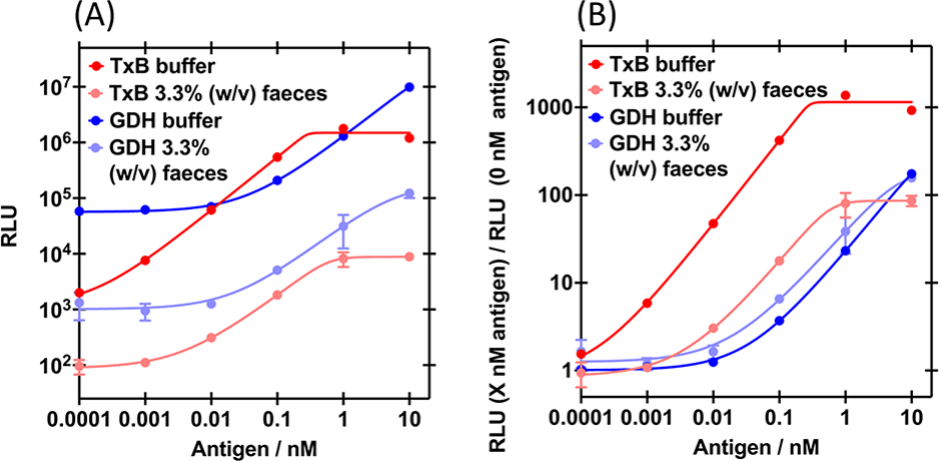
Effect of feces on the split-luciferase assay dose response curves. (A) Bioluminescence and (B) fold gain in bioluminescence of 0.5 nM S-E3 + 1 nM L-45 or 8 nM 4-S + 8 nM 4-L, with TxB or GDH, respectively, in the presence of 0 or 3.33% w/v feces. Sensor proteins (final concentration in the assay indicated) and analyte (final concentration in the assay indicated) incubated with *C. difficile* negative fecal sample or buffer for 30 min, at 25 °C, with agitation. Nano-Glo added to a final concentration of 1:1000 and bioluminescence read after 2 min. Data are the mean of duplicates on the same plate, error bars indicate standard deviation from the mean and solid lines are 5PL regression fits (A: 0.939 < *R*^2^ < 0.993 and B: 0.939 < *R*^2^ < 1.000).

While the assays are functional with stool, there are significant fecal sample matrix effects. For the TxB assay, increasing the concentration of stool from 0.0067–3.33% w/v decreases both raw and fold gain in bioluminescence (Figure S14A,B) and increases signal loss over time (Figure S15). Nevertheless, the results indicate that the greatest sensitivity would be obtained with 3.33% w/v stool, as for real samples further dilution would reduce the amount of TxB more than is compensated for by reduced matrix effects (Figures S14C and S16A). At even higher stool concentrations, the matrix effect then becomes too detrimental (Figure S16A). The 2 pM LoD in 3.33% w/v stool (Figure S16A) is significantly higher than the 44 fM LoD in buffer, but comparable to the 0.16 ng mL^–1^ = 0.6 pM cut off of Quik Chek (also in 3.33% w/v stool matrix; Figure S16B).^27^ For the GDH assay, 3.33% w/v feces reduces raw bioluminescence (Figure 5A) and increases signal loss over time (Figure S17). Fold gain in bioluminescence with GDH is much less affected by 3.33% w/v feces (Figure 5B) and the 4.5 pM LoD is comparable to the 0.8 ng mL^–1^ = 3 pM cut off of Quik Chek.^27^ The sensitivity and time-to-result (approximately 30 min) of our GDH/TxB stool tests are comparable to this current POCT, but less user steps (e.g., washing) are required and results are quantitative rather than qualitative. It may provide an important research tool to further investigate correlation of the fecal toxin levels and disease severity for prognosis and therapy guidance.^28^

Current clinical diagnostics for CDI have major limitations,^8,29^ especially in differentiating the disease-free carriage of *C. difficile* from true CDI with toxin production.^3,29^ This leads to poor treatment and inappropriate antibiotic prescribing that, in fact, increases CDI and drug-resistant infection risk by dysbiosis.^2,3^ The reference test for true CDI (cell cytotoxity neutralization assay, CCNA) sensitively detects toxin but is too slow and complex for routine use.^29,30^ Faster nucleic acid amplifications tests (NAATs) detect toxin genes, but not expression so lack clinical specificity, while EIAs and LFTs detect free toxin but lack the sensitivity of CCNA.^9,31^ No one test is sufficient and clinical guidance relies on multistep algorithms, leading to delays and confusion with discordant results.^8,29^ Recent ultrasensitive toxin immunoassays offer promise as standalone tests for true CDI.^32^ However, commercial “single molecule counting” technology relies on specialized instrumentation for magnetic separation and fluorescent imaging detection,^33,34^ while electrochemical sandwich assays require multiple incubation and wash steps.^35^ Our homogeneous wash-free assay is much simpler and amenable to adaptation into a POCT. The 12 pg mL^–1^ = 44 fM TxB LoD and 4–5-log range in buffer compares favorably with clinically relevant concentrations and the ∼20 pg mL^–1^ = 74 fM cutoff for optimal sensitivity and specificity versus CCNA.^33^ Recently described POCTs based on a paper device and label-free electrochemical sensing have lower sensitivity and range, respectively.^36^ Therefore, if our assay can be adapted into a POCT and fecal sample matrix effects reduced, it will offer an urgently required improvement for ultrasensitive toxin detection and CDI diagnosis. Clinical TxB concentrations of CDI samples have an upper limit of 100 ng mL^–1^ with some samples going up to 1,000 ng mL^–1^ (370 pM and 3.7 nM, respectively).^33^ At these highest TxB concentrations (>1 nM TxB), the reading of the assay levels off and quantitative determination of the TxB concentration will be less accurate. If a quantitative concentration determination at >1 nM TxB is required, the sample can be diluted prior to the assay.

Fecal matter absorbs light, reducing the bioluminescent signal. It could also inhibit the luciferase, break down the substrate, and nonspecifically bind or degrade the sensor proteins and analyte. The exact mechanisms of such effects on the TxB and GDH assays are unclear, but feces are certainly a complex matrix and detrimental to both assays. Stool samples are also heterogeneous, so effects are dependent on the exact patient sample (Figure S18). In future development of a POCT, the centrifugation step will need to be replaced. To improve assay performance and consistency, a POCT could have a filter, include additives to reduce matrix effects, and be performed in a thin layer (e.g., paper device) to reduce absorption of light. All assay components could be freeze-dried on a paper device to give a POCT that simply requires sample addition and measurement with a luminometer or camera.^14,25^ Improving fundamental assay sensitivity would also allow greater stool sample dilution, further reducing feces matrix effects. The four TxB binding proteins (Affimers 18 and 45, nanobodies E3 and 7F) bind different epitopes, so two could be fused to LgBiT and the other two to SmBiT, to enhance binding affinity by avidity effects and potentially increase assay sensitivity. To measure TxB and GDH in parallel, two tests would have to be performed in the POCT or the wavelength of the two NanoBiT sensors could be tuned to enable two simultaneous measurements from a single test.^37^

## Conclusions

We have selected and characterized Affimers (13 kDa nonimmunoglobulin binding proteins) targeting *C. difficile* biomarkers GDH and TcdB, which can be used in diagnostic assays for CDI. When incorporated alongside nanobodies (single domain antibodies) as binding proteins in NanoBiT split-luciferase assays, they show advantages over antibodies in terms of ease of production and potentially assay performance. We expect that these NanoBiTBiP (NanoBiT with Binding Proteins) assays will be generally applicable to other biomarkers and offer a promising underlying technology for POCTs.

The NanoBiTBiP assays for GDH and TxB were optimized and factors controlling assay performance, including binding protein affinity, sensor design, sensor concentration, incubation times and substrate concentration, were examined. Intra- and interassay sensitivity, accuracy, and precision metrics were established and the 1300-fold signal gain, 44 fM LoD and 4–5 log range of the TcdB assay is the best observed for a NanoBiT assay. When the GDH and TxB assays are performed in 3.33% (w/v) feces there is a significant fecal sample matrix effect but sensitivity (LoD = 4.5 and 2 pM, respectively) and time to result (32 min) are comparable to the currently used point-of-care LFT, Quik Chek Complete. Our homogeneous NanoBiTBiP assay has less user steps (e.g., washing), specifically detects the more clinically relevant TcdB (not TcdA), and is quantitative. It may offer an important research tool to investigate the correlation of fecal TcdB concentration and clinical outcomes to improve therapy guidance.

An ultrasensitive TcdB assay holds promise as an urgently required standalone test that, unlike current diagnostics, is able to accurately distinguish *C. difficile* carriage from true CDI with toxin production. This would ensure timely effective treatment for CDI and avoid inappropriate antibiotic prescribing for carriage, which actually increases CDI and drug-resistant infection risk. Future research should focus on adapting the TcdB NanoBiTBiP assay into an ultrasensitive POCT that will reduce the heavy burden of CDI on patients and healthcare systems.

## Supporting Information Available

Detailed experimental methods of Affimer selection, validation, characterization by SPR, and sensor cloning; Modeling; Supplementary figures (Figures S1–S18); DNA and protein sequences; Tables of primers (PDF)
