# Supplementary material for: Rapid Quantification of C. difficile Glutamate Dehydrogenase and Toxin B (TcdB) with a NanoBiT Split-Luciferase Assay
Source: Anal Chem. 2022 May 28;94(23):8156–63. doi: 10.1021/acs.analchem.1c05206 (PMC9201815; doi:10.1021/acs.analchem.1c05206)
Supplement: Supplementary file 1 — ac1c05206_si_001.pdf [file ac1c05206_si_001.pdf]

Supplementary Information

Rapid Quantification of *C. difficile* Glutamate Dehydrogenase (GDH) and Toxin B (TcdB) with a NanoBiT Split-Luciferase Assay

Hope Adamson[a], Modupe O. Ajayi[b], Kate E. Gilroy[b], Michael J. McPherson[b], Darren C. Tomlinson[b,*] and Lars J. C. Jeuken[a,c,*]

[a] School of Biomedical Sciences and Astbury Centre for Structural Molecular Biology, University of Leeds, Leeds, LS2 9JT, United Kingdom;

[b] School of Molecular and Cellular Biology and Astbury Centre for Structural Molecular Biology, University of Leeds, Leeds, LS2 9JT, United Kingdom;

[c] Leiden Institute of Chemistry, Leiden University, PC Box 9502, 2300 RA, Leiden, the Netherlands;

[*] Corresponding authors: Darren C. Tomlinson, D.C.Tomlinson@leeds.ac.uk and Lars J.C. Jeuken, L.J.C.Jeuken@lic.leidenuniv.nl

Table of Contents

Experimental Methods....S-2
Modelling....S-4

Supplementary Figures and Tables
Figure S1: Schematic representation of the complexes in the NanoBiT split-luciferase assay. S-4
Figure S2. Selection of TcdB Affimers S-6
Figure S3. Selection of GDH Affimers....S-6
Figure S4. SPR binding curves....S-7
Figure S5. SDS-PAGE of split NanoLuc sensor proteins....S-8
Figure S6: Thermodynamic model of the TxB split NanoLuc assay....S-8
Figure S7. Establishing optimal TxB sensor protein concentrations S-9
Figure S8. Kinetics of the TxB split NanoLuc assay....S-9
Figure S9. Kinetics of the GDH split NanoLuc assay S-10
Figure S10. Establishing optimal Nano-Glo substrate concentration S-11
Figure S11. Optimised dose response curves used to calculate intra-assay (A, B) and inter-assay (C, D, E, F) LoD, accuracy (% recovery) and precision (% CV)....S-12
Figure S12. Fold gain response of the TxB split-luciferase assay in faecal sample matrix....S-13
Figure S13. Effect of sensor protein concentrations on TxB dose response in faecal sample matrix....S-13
Figure S14. Effect of % faecal sample matrix on the TxB dose response S-14
Figure S15. Effect of % faecal sample matrix on the TxB split-luciferase assay over time....S-14
Figure S16. Measurement of the limit of detection (LoD) of the TxB split-luciferase assay vs. C. diff Quik Chek complete test S-15
Figure S17. Effect of faeces on the TxB and GDH split-luciferase assays over time....S-15
Figure S18. Effect of faecal samples from two different patients on the TxB split-luciferase assay....S-16
Table S1. Sensitivity (LoD), Accuracy (% recovery) and Precision (% CV) of TxB and GDH assays S-16

DNA and Protein Sequences of Affimers, Nanobodies and LgBiT....S-17
DNA and Protein Sequences of the Sensor constructs....S-19
Tables of Primers....S-21
References S-22

Experimental Methods

Affimer Selection and Validation

Targets

C. difficile GDH was produced recombinantly and used for all Affimer selection and validation work. Codon optimised synthetic DNA encoding *C. difficile* GDH (Genbank M65250) was purchased from Genscript. This was subcloned into a pET28c expression vector by restriction enzyme cloning between *NcoI* and *HindIII* sites, with an in-frame c-terminal 6-Histag. Following transformation into *E. coli* BL21* (DE3) cells, a 2 ml starter culture was added to 50 ml LB media (with 50 µg ml^{-1} kanamycin) and grown at 37°C, 230 RPM before induction at OD_{600} ca. 0.8 with 0.1 mM isopropyl-β-D-thiogalactoside (IPTG) and 6 hours growth at 25°C, 150 RPM. Cells were harvested at 4000 *g* for 15 min and lysed by resuspension in 1 ml lysis buffer (50 mM NaH_2PO4, 300 mM NaCl, 20 mM imidazole, pH 7.4 supplemented with 100 µl BugBuster 10X Protein Extraction Reagent (Novagen), 0.4 µl Benzonase Nuclease (Novagen) and 10 µl of Halt Protease Inhibitor cocktail EDTA-Free (100X) (Thermo Scientific)). Insoluble material was pelleted by centrifugation at 17000 *g* for 20 min. The supernatant was added to 300 µl Ni-NTA resin slurry (Qiagen) pre-equilibrated with wash buffer (50 mM NaH_2PO4, 500 mM NaCl, 20 mM imidazole, pH 7.4) and incubated on a roller mixer for 1.5 hour at room temperature. Resin was washed with wash buffer until the A_{280} of the wash fraction was < 0.01, then protein was eluted with 0.5 ml fractions of elution buffer (50mM NaH_2PO4, 500 mM NaCl, 300 mM imidazole, pH 7.4). Pure fractions (assessed by SDS-PAGE) were dialysed into PBS, protein concentration measured by A_{280} and aliquots stored at -80°C. GDH from *C. difficile* (List Biologicals Laboratories) was used for SPR and sensor characterisation work.

Native *C. difficile* toxins A and B (VPI 10463 strains) were provided by Dr Cliff Shone, Public Health England (PHE), Porton Down and used for all Affimer selection and validation work. Inactivated toxoids derived from purified *C. difficile* toxins A and B (Biorad) were used for SPR and sensor characterisation work. Toxins A and B were biotinylated using EZ-Link NHS-SS-biotin (Thermo Fisher) and GDH with EZ-Link HPDP-Biotin (Thermo Fisher), according to the manufacturer's instructions.

Phage display

Phage display screening of the Affimer library was performed as previously described,[1] with adjustments described as follows. Streptavidin coated wells (Pierce) were incubated with biotinylated target for 2 hours, washed with PBST then incubated with pre-panned phage for 2.5 hours. Panning wells were washed with PBST, eluted with 200 mM glycine–HCl (pH 2.2) for 10 min, neutralised with 1 M Tris–HCl (pH 9.1), further eluted with 100 mM triethylamine for 6 min and neutralised with 1 M Tris–HCl (pH 7). ER2738 cells were infected with eluted phage for 1 hour at 37°C, 90 rpm and were then plated onto LB agar (with 100 µg/ml carbenicillin) for overnight growth at 37°C. Colonies were scraped into 8 ml 2TY (with 100 µg/ml carbenicillin) to a dilution of A_{600} = 0.2, incubated for 1 hour at 37°C and 230 rpm, infected with M13K07 helper phage and grown for 30 mins at 37°C, 90 rpm. Then 25 µg/ml kanamycin was added prior to overnight incubation at 25°C, 170 rpm. Phage were precipitated with 4 % (w/v) PEG 8000, 0.5 M NaCl and resuspended in 320 µl 10 mM Tris, pH 8.0, 1 mM EDTA (TE buffer). For panning round two, streptavidin magnetic beads (Dynabeads MyOne Streptavidin T1, Invitrogen) were incubated with biotinylated target for 1 hour, washed and then incubated with pre-panned phage for 1 hour. They were then washed four times using a KingFisher instrument (Thermo Fisher), before elution and amplification of the phage as described above. The final pan was as described for panning round one but using Neutravidin coated wells (Pierce). For the toxin B screen there was an additional 24 hour incubation with toxin A and wash step prior to phage elution, in order to remove cross-reactive phage.

Phage ELISA

Individual colonies from the target plates were randomly selected and grown overnight in 200 µl of 2TY (with 100 µg/ml carbenicillin) in a 96-deep well plate at 37°C, 1050 rpm. Then 200 µl of 2TY (with 100 µg/ml carbenicillin) was inoculated with 25 µl culture and grown at 37°C, 1050 rpm for 1 hour. Helper phage (10 µl of 10^{11}/ml) were added and kanamycin to 25 µg/ml, prior to overnight incubation at room temperature, 750 rpm. The culture was then centrifuged at 3500 *g* for 10 min and the supernatant used for phage ELISA. In the wells of a F96 Maxisorp Nunc-immuno plate, 50 µl of 2.5 µg/ml streptavidin in PBS was incubated overnight at 4°C or for 2 hours at room temperature. Streptavidin coated wells were then blocked with 200 µl 2 x casein blocking buffer (Sigma) overnight at 37°C, washed with 300 µl PBST and then incubated with 50 µl biotinylated target or controls for 1 hour at room temperature with agitation. Wells were washed with 300 µl PBST prior to addition of 10 µl casein blocking buffer, 40 µl supernatant phage and incubation for 1 hour at room temperature with agitation. Wells were then washed with 300 µl PBST, prior to addition of 50 µl 1:1000 HRP conjugated anti-Fd-Bacteriophage (Seramun) and incubation for 1 hour at room temperature with agitation. Wells were then washed 10x with 300 µl PBST before addition of 50 µl TMB (Seramun) and absorbance read at 620 nm.

Affimer Expression and Purification

Affimer DNA coding sequences were PCR amplified and subcloned into pET11a expression vector by restriction enzyme cloning between *NheI* and *NotI* sites, with an in-frame c-terminal 8-Histag. Appropriate primers were used to introduce a cysteine codon prior to the Histag when required for site-specific biotinylation. Protein production was exactly as described for *C. difficile* GDH, with expression in *E. coli* BL21* (DE3) cells and purification with Ni-NTA resin. Affimers with a c-terminal cysteine residue were biotinylated with EZ-Link HPDP-Biotin (Thermo Scientific), according to the manufacturer's instructions.

ELISA

Affimer (or target) at 10 μg/ml in PBS was absorbed onto Immuno 96 Microwell Nunc Maxisorp plate wells overnight at 4°C. Wells were blocked with 200 µl of 3 × casein blocking buffer (Sigma) at 37°C for 4 hours. Biotinylated target (or Affimer) was adsorbed at 1 μg/ml in 2 × casein blocking buffer for 1 hour with shaking. Detection was with 1:1000 streptavidin conjugated HRP (Invitrogen) in PBST, added for 1 hour. After washing with PBST, 50 µl TMB (Seramun) was added and absorbance read at 620 nm.

Sandwich ELISA

50 µl of Affimer at 10 µg/ml in 2 x casein blocking buffer (Sigma) was incubated in Immuno 96 Microwell Nunc Maxisorp wells overnight at 4°C with gentle agitation. Wells were blocked with 200 µl of 2 x casein blocking buffer for 4 hours at 37°C, washed once with 300 µl PBST and then 50 µl of 10 µg/ml target added and incubated for 1 hour at room temperature with gentle agitation. Wells were washed once with 300 µl PBST, then biotinylated Affimer at 10 µg/ml in 2 × casein blocking buffer was added and incubated for 1 hour with shaking. Wells were washed three times with PBST then 50 µl 1:1000 streptavidin conjugated HRP (Invitrogen) added for 1 hour. After washing with PBST, 50 µl TMB (Seramun) was added and absorbance read at 620 nm.

Surface Plasmon Resonance (SPR)

Affimers and nanobodies (with N-terminal SmBiT101) were biotinylated using EZ-link-NHS-PEG4-biotin (Thermo Fisher Scientific) using a 1:1 molar ratio and 30 min incubation at room temperature. Kinetic binding analysis was performed with a BIAcore T200 instrument (Cytiva), at 25°C and with PBS + 0.05 % Tween 20 running buffer. A streptavidin (SA) sensor chip was conditioned with three 1 min injections of 1M NaCl, 50 mM NaOH at 40 µl min^{-1}. Flow cell 1 was an unmodified reference, whilst flow cells 2-4 had biotinylated Affimer or nanobody immobilised at 5 µl min^{-1} to the following ligand densities (Affimer 4, ~ 80 RU; Affimer 18, ~ 45 – 80 RU; Affimer 45, ~ 1250 RU; Nanobody E3, ~ 200 RU; Nanobody 7F, ~ 400 RU). Following equilibration with running buffer serial dilutions of GDH, TxA or TxB analyte were injected at 30 µl min^{-1} for 3 min, followed by 10 min dissociation. The surface was regenerated with a 30 s injection of 0.1 M Na_2CO_3. Data were normalised by subtraction of responses from the unmodified reference cell and a buffer only injection. The association (k_a) and dissociation (k_d) rate constants and equilibrium dissociation constant (K_D) were determined from a global fit to the SPR curves with a 1:1 Langmuir model using the BIAevaluation software.

Sensor Cloning

DNA and primers (Integrated DNA Technologies) used are detailed in the "DNA and Protein Sequences" and "Tables of Primers" sections, respectively. All sensor constructs were generated in a pET28a vector containing *NheI*, *NotI*, *SpeI* and *SalI* restriction sites between the *NcoI* and *XhoI* sites of the vector, with an in frame 6xHistag sequence and stop-codon following *XhoI*. Sequential restriction enzyme cloning was used to insert DNA encoding LgBiT, SmBiT (101), Affimer or nanobody sequences between *NheI*/*NotI* and *SpeI*/*SalI* and a $(GSG)_7$ linker sequence between *NotI* and *SpeI*. The vector was digested with appropriate restriction enzymes (NEB), dephosphorylated with antarctic phosphatase (NEB), separated on an agarose gel and then purified. All DNA was purified using the Illustra GFX PCR DNA and Gel Band Purification Kit (GE Healthcare). The synthetic DNA encoding LgBiT and nanobodies E3 and 7F was purchased from Genscript in pUC57 vectors. Affimers 18, 28 and 4 were encoded in the pET11a vectors described in the "Affimer Selection and Validation" section. This insert DNA was PCR amplified with primers encoding appropriate restriction sites, then treated with *DpnI* (NEB) to remove parental vector DNA. Insert DNA encoding SmBiT (101) and $(GSG)_7$ linker sequences were generated by PCR of overlapping primers encoding appropriate restriction sites. Amplified insert DNA was purified, digested with appropriate restriction enzymes and then re-purified. The digested vector and insert were ligated with T4 DNA ligase (NEB) and transformed into *E. coli* XL-1 competent cells (Agilent Technologies). Plasmid DNA was purified using the ChargeSwitch Pro Plasmid Miniprep Kit (Invitrogen) and successful generation of constructs was confirmed by sequencing (Genewiz) with T7 / T7term primers.

Modelling

To understand the response of the NanoBiT sensors, bioluminescence was modelled. We adopted the thermodynamic model described by Ni et al.[2] This thermodynamic model assumes the binding affinities between the analyte and binding proteins (Affimers or Nanobodies) are not affected by N-terminal or C-terminal addition of SmBiT101 or LgBiT fragments. The model further assumes that the epitopes of the binding proteins do not overlap and binding of both binding proteins are thus independent and described by the K_d determined by SPR (Table 1 of the main manuscript). The full model is described in the scheme below, with * indicating the active NanoBiT enzyme:

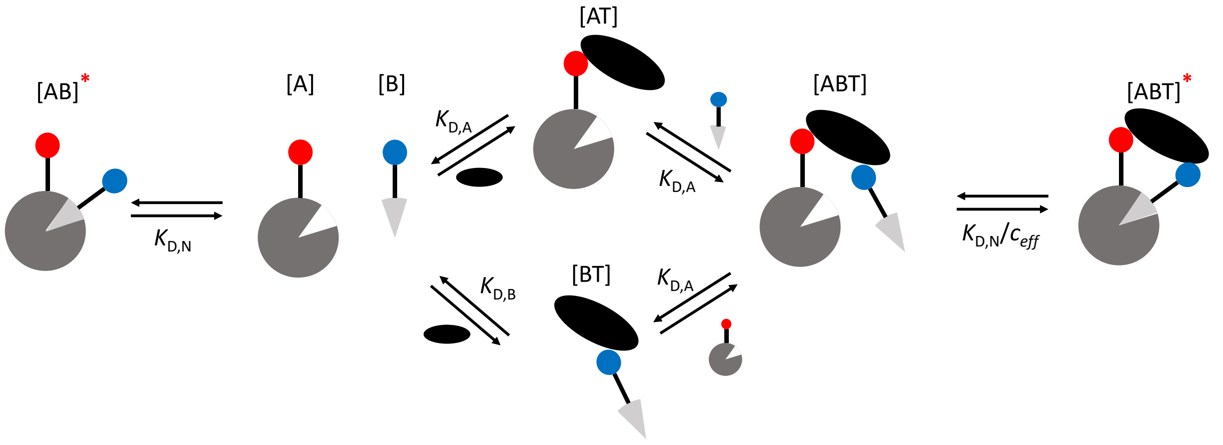

Figure S1: Schematic representation of the complexes in the NanoBiT split-luciferase assay. The small (SmBiT, light grey, B) and large (LgBiT, dark grey, A) of the NanoBiT split-luciferase are coupled to two binding proteins (red and blue). The binding to the analyte (T, black) is indicated. The two species indicated with a red asterisk are active and hence lead to a bioluminescence output.

The total amounts of binding proteins ($[A]_0$ and $[B]_0$) and analyte ($[T]_0$) is given by:

$$[A]_0 = [A] + [AB]^* + [AT] + [ABT] + [ABT]^* \quad \text{(Eq. S1)}$$

$$[B]_0 = [B] + [AB]^* + [BT] + [ABT] + [ABT]^* \quad \text{(Eq. S2)}$$

$$[T]_0 = [T] + [AT] + [BT] + [ABT] + [ABT]^* \quad \text{(Eq. S3)}$$

The binding equilibria are given by:

$$K_{D,A} = \frac{[A][T]}{[AT]} \quad \text{(Eq. S4)}$$

$$K_{D,B} = \frac{[B][T]}{[BT]} \quad \text{(Eq. S5)}$$

$$K_{D,N} = \frac{[A][B]}{[AB]^*} \quad \text{(Eq. S6)}$$

$$K_{D,A} = \frac{[BT][A]}{[ABT]} \quad \text{(Eq. S7)}$$

$$K_{D,B} = \frac{[AT][B]}{[ABT]} \quad \text{(Eq. S8)}$$

$$K_{D,N} = \frac{[ABT]c_{eff}}{[ABT]^*} \quad \text{(Eq. S8)}$$

Combining equations S1-S8 gives

$$[A]_0 = [A] + \frac{[A][B]}{K_{D,N}} + \frac{[A][T]}{K_{D,A}} + \frac{[A][B][T]}{K_{D,A}K_{D,B}} + \frac{[A][B][T]c_{eff}}{K_{D,A}K_{D,B}K_{D,N}} \quad \text{(Eq. S9)}$$

$$[B]_0 = [B] + \frac{[A][B]}{K_{D,N}} + \frac{[B][T]}{K_{D,B}} + \frac{[A][B][T]}{K_{D,A}K_{D,B}} + \frac{[A][B][T]c_{eff}}{K_{D,A}K_{D,B}K_{D,N}} \quad \text{(Eq. S10)}$$

$$[T]_0 = [T] + \frac{[A][T]}{K_{D,A}} + \frac{[B][T]}{K_{D,B}} + \frac{[A][B][T]}{K_{D,A}K_{D,B}} + \frac{[A][B][T]c_{eff}}{K_{D,A}K_{D,B}K_{D,N}} \quad \text{(Eq. S11)}$$

Equation S9-S11 were solved using Matlab (R2021b). The function *fsolve* was used to solve [A], [B] and [T] for a set $[A]_0$, $[B]_0$, $[T]_0$ values and $K_{D,A}$ and $K_{D,B}$ values as given in Table 1 of the main manuscript (for a given set of binding proteins). $K_{D,N}$ between SmBiT and LgBiT was previously determined to be 2.5 μM.[2, 3] Using these values, $[AB]^*$ and $[ABT]^*$ are calculated from Eq. S6 and S8, respectively, and the fold increase in bioluminescence (RLU) at a given $[T]_0$ is given by:

$$\mathrm{RLU}_{\mathrm{fold\ increase}} = \frac{[\mathrm{ABT}]^*_{\{T]_0} + [\mathrm{AB}]^*_{\{T]_0}}{[\mathrm{AB}]^*_{[T]_0=0}} \qquad \text{(Eq. S12)}$$

The MatLab code is run by calling the Matlab code file **ABT_star.m** in the command window after coding the following two code files (equation F(1), F(2) and (F3) are S9, S10 and S11 above). Note that the values in these code files are in nM.

Massbalance.m

```
function F = balance(x,T0,A0,B0,KN,KA,KB,EM) %x(1) = A; x(2) = B; x(3) = T
F(1) = -A0 + x(1) + x(1)*x(2)/KN + x(1)*x(3)/KA + x(1)*x(2)*x(3)/(KA*KB) + (1)*x(2)*x(3)*EM/(KA*KB*KN)
F(2) = -B0 + x(2) + x(1)*x(2)/KN + x(2)*x(3)/KB + x(1)*x(2)*x(3)/(KA*KB) + (1)*x(2)*x(3)*EM/(KA*KB*KN)
F(3) = -T0 + x(3) + x(1)*x(3)/KA + x(2)*x(3)/KB + x(1)*x(2)*x(3)/(KA*KB) + (1)*x(2)*x(3)*EM/(KA*KB*KN)
end
```

ABT_star.m

```
clear RLU %this fold gain of RLU
clear TxB

A0=2; %concentration of total binding protein A in nM
B0=2; %concentration of total binding protein B in nM
KN=2.5e3; %KD,N in nM
KA=2.5; %KD,A in nM, E3
KB=13; %KD,B in nM, 45

EM=1e6; %c(eff) in nM (= 1 mM)

j=0;

T0=0; %concentration of total analyte in nM
x0=[0,0,0]; %x(1) = A; x(2) = B; x(3) = T
F=@(x)massbalance(x,T0,A0,B0,KN,KA,KB,EM);
x=fsolve(F,x0);
blank=x(1)*x(2)/KN;

for i=-4:0.1:1
T0=10^(i); %concentration of total analyte in nM
x0=[10,10,10]; %starting parameters for the Levenberg-Marquardt algorithm; might need optimisation
F=@(x)massbalance(x,T0,A0,B0,KN,KA,KB,EM);
x=fsolve(F,x0);

j=j+1;
TxB(j)=T0;
RLU(j)=(4*x(1)*x(2)*x(3)*EM/(KA*KB*KN) + x(1)*x(2)/KN)/blank; %Eq. S12, this fold gain of RLU
end

save('Model output.txt','TxB','RLU','-ascii')
loglog(TxB,RLU)
```

Supplementary Figures

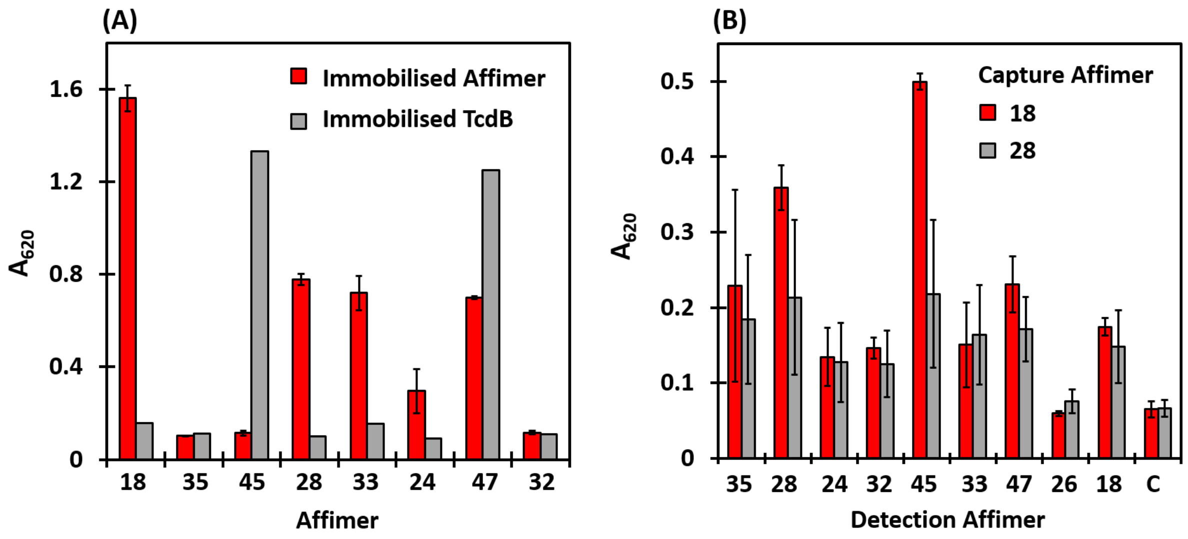

Figure S2. Selection of TcdB Affimers. (A) ELISA to assess binding of adsorbed Affimer (red) or TcdB (grey) to biotinylated TcdB or Affimer, respectively. (B) Sandwich ELISA to assess pairwise binding of adsorbed "capture Affimer" 18 (red) or 28 (grey) and biotinylated "detection Affimer" (including non-binding control Affimer C) with TcdB. For both ELISA and sandwich ELISA, detection was with streptavidin-HRP and visualisation with TMB, read at 620 nm. Where error bars are present, data are the mean of duplicate measurements on the same plate and error bars indicate the standard deviation.

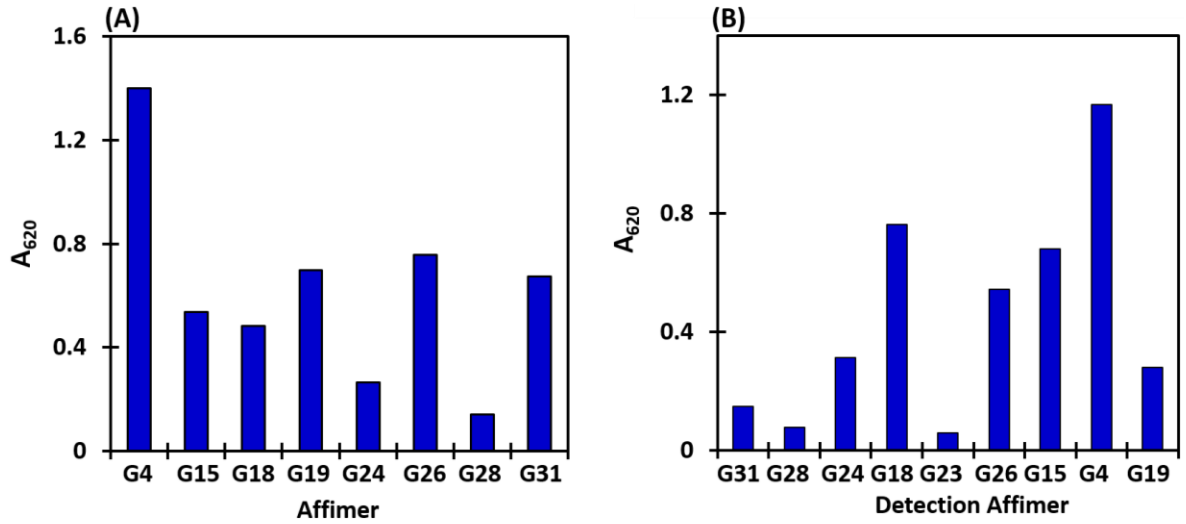

Figure S3. Selection of GDH Affimers. (A) ELISA to assess binding of adsorbed Affimers to biotinylated GDH (B) Sandwich ELISA to assess pairwise binding of adsorbed Affimer G4 (referred to as Affimer 4 in the main text) and biotinylated "detection Affimer" with GDH. For both ELISA and sandwich ELISA, detection was with streptavidin-HRP and visualisation with TMB, read at 620 nm.

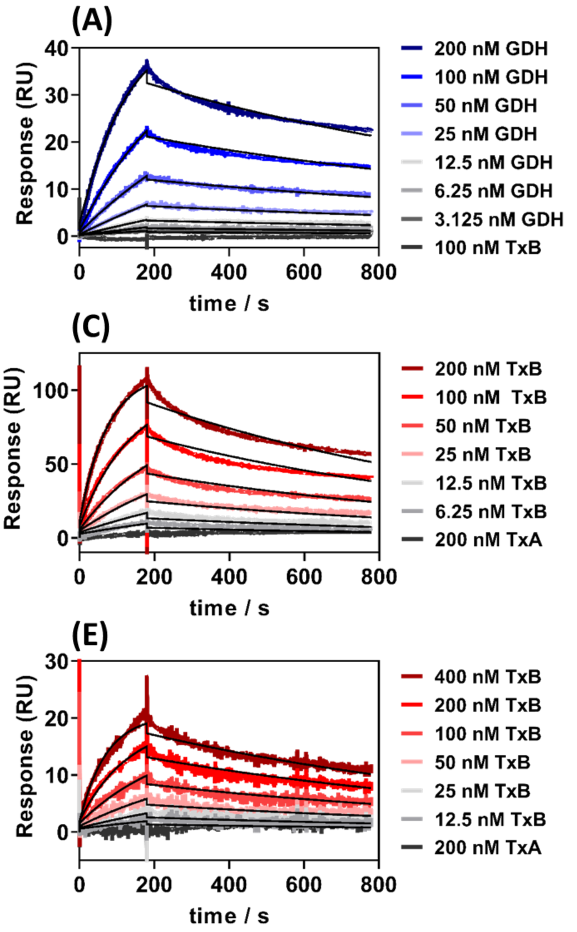

Figure S4. SPR binding curves for (A) Affimer 4 (B) Affimer 18 (C) Affimer 45 (D) Nanobody E3 and (E) Nanobody 7F. Affimers / nanobodies were biotinylated and immobilised on a streptavidin chip. Serial dilutions of analyte injected at 30 µl min^{-1} for 3 min, followed by 10 min dissociation. Data normalised by subtraction of responses from unmodified reference cell and buffer only injection. A global fit to the SPR curves (black line) was made with a 1:1 Langmuir model using the BIAevaluation software and used to determine the association (k_a) and dissociation (k_d) rate constants and equilibrium dissociation constant (K_D).

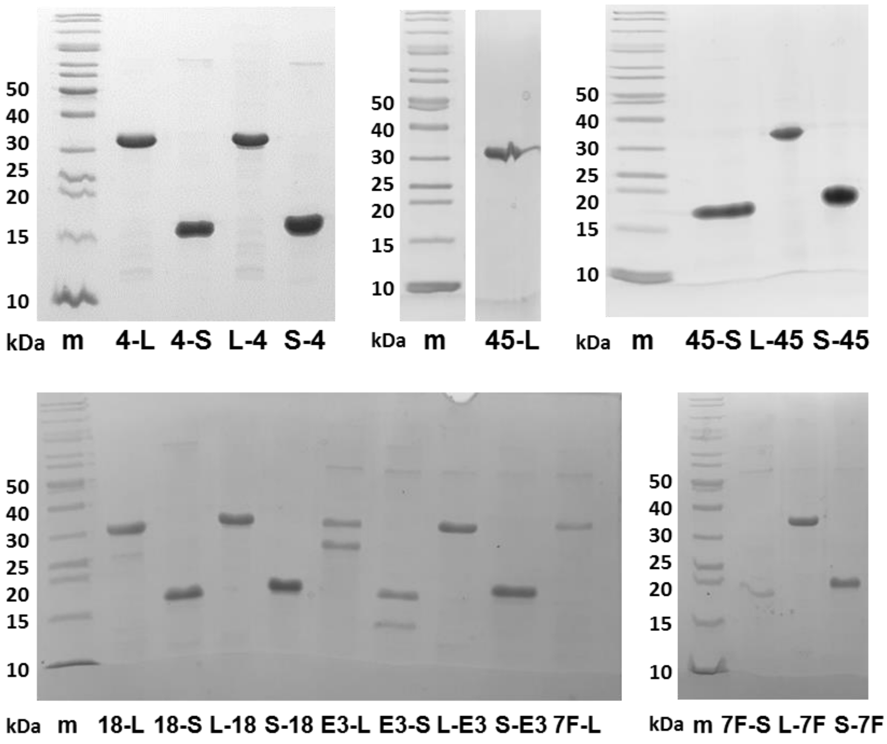

Figure S5. SDS-PAGE of split NanoLuc sensor proteins

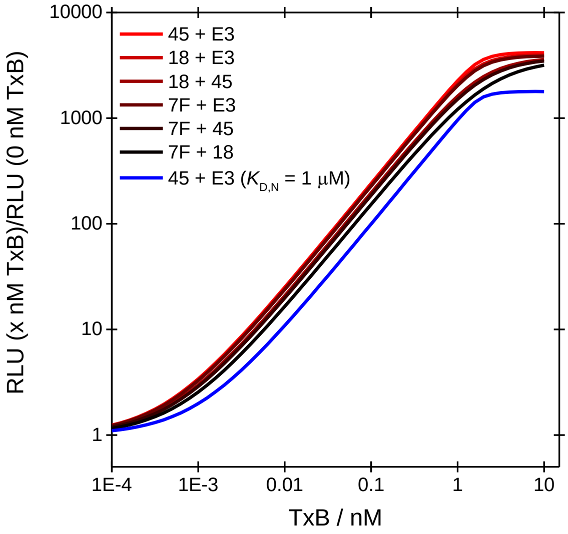

Figure S6: Thermodynamic model of the TxB split NanoLuc assay. The model is described in the Experimental Methods section. The model has the following parameters: Effective concentration c_{eff} = 1 mM, $K_{D,A}$ and $K_{D,B}$ for the nanobodies and Affimers, as indicated in the legend, were taken from Table 1. $K_{D,N}$ = 2.5 μM (except Blue line for which $K_{D,N}$ = 1 μM). Sensor proteins, $[A]_0$ and $[B]_0$ = 2 nM. TxB, $[T]_0$ as indicated.

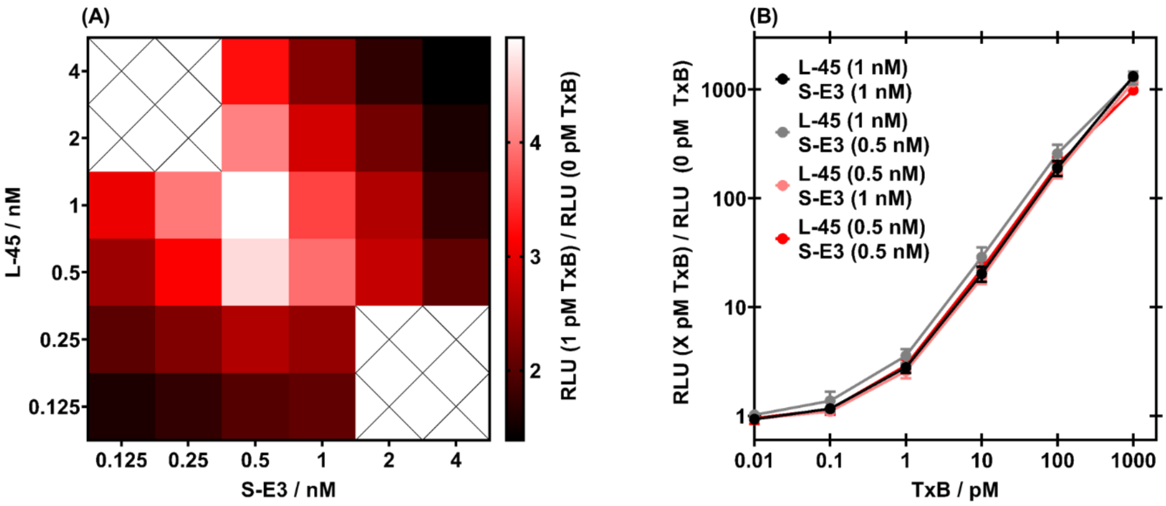

Figure S7. Establishing optimal TxB sensor protein concentrations (A) Heat map of fold gain in bioluminescence of 0.125 – 4 nM S-E3 + 0.125 – 4 nM L-45 with 1 pM TxB (B) Dose response of 0.5 – 1 nM S-E3 + 0.5 – 1 nM L-45 with TxB. Data are the mean of three independent measurements and error bars indicate standard deviation from the mean. For all assays, analyte (final concentration indicated) and sensor proteins (final concentration indicated) were incubated for 30 mins, at 25°C, with agitation prior to addition of Nano-Glo substrate to a final dilution of 1:1000 and bioluminescence was immediately read.

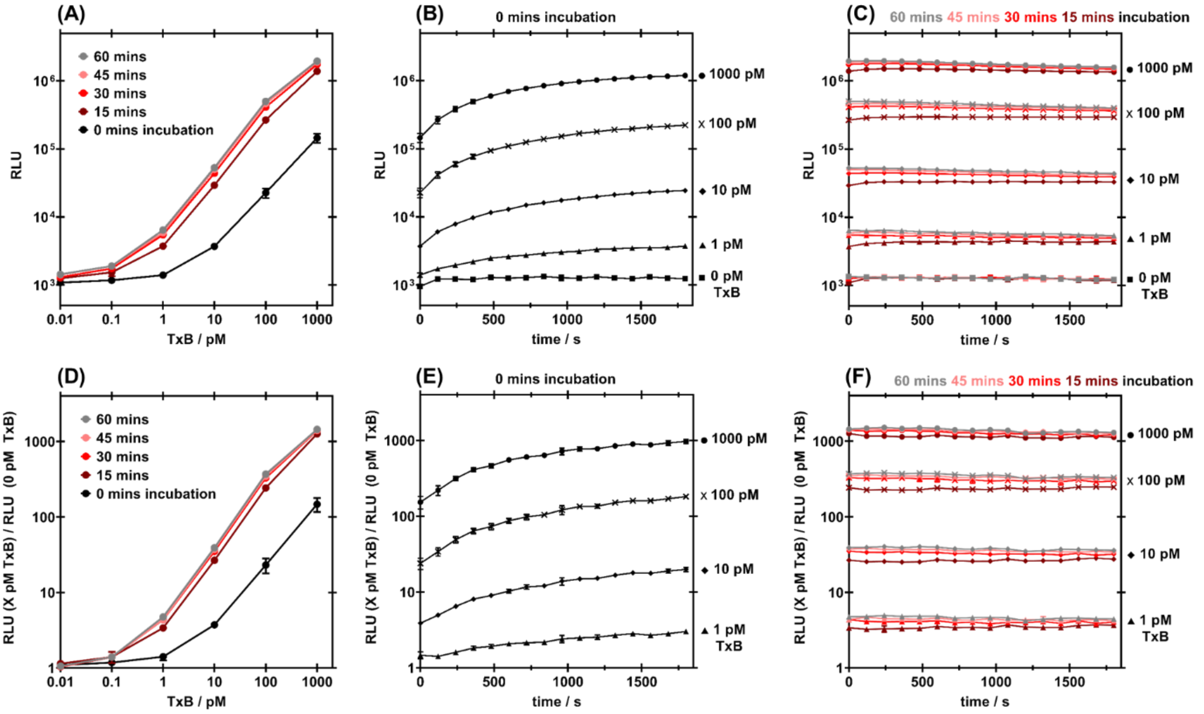

Figure S8. Kinetics of the TxB split NanoLuc assay (A) Bioluminescence and (D) Fold gain in bioluminescence dose response curve, read immediately after substrate addition following a 0-60 mins pre-incubation. (B) Bioluminescence and (E) Fold gain in bioluminescence *vs.* 0 pM TxB over time after no pre-incubation. (C) Bioluminescence and (F) Fold gain in bioluminescence *vs.* 0 pM TxB over time after 15-60 mins pre-incubation. For all assays, TxB (final concentration indicated), S-E3 (0.5 nM final concentration) and L-45 (1 nM final concentration) were incubated for the indicated length of time, at 25°C, with agitation prior to addition of Nano-Glo substrate to a final dilution of 1:1000. Data are the mean of duplicates on the same plate and error bars indicate standard deviation from the mean.

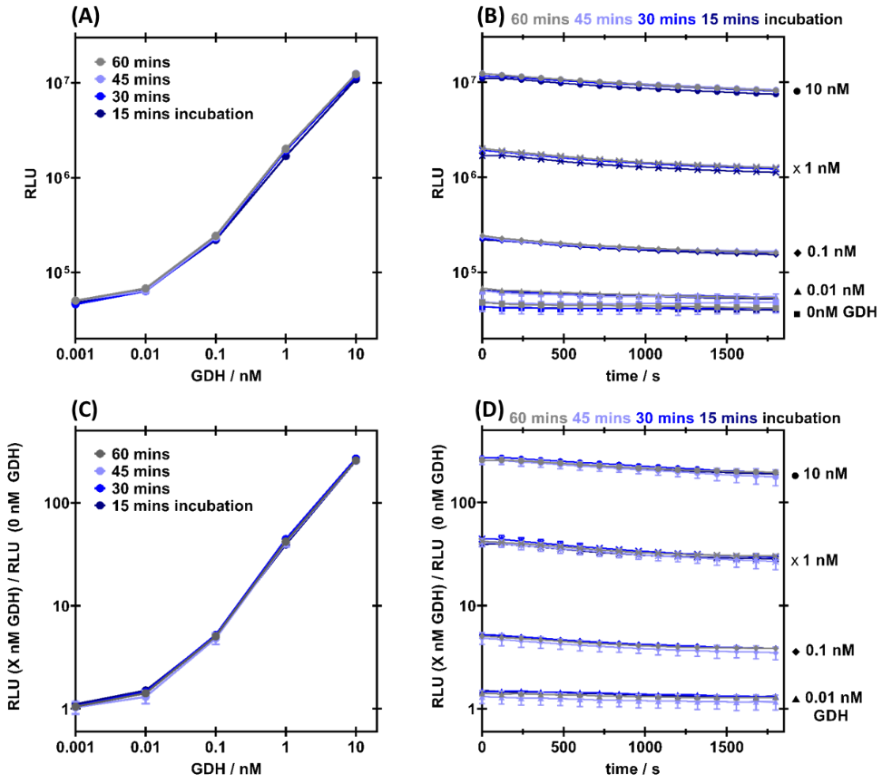

Figure S9. Kinetics of the GDH split NanoLuc assay (A) Bioluminescence and (C) Fold gain in bioluminescence dose response curve, read immediately after substrate addition following a 15-60 mins pre-incubation. (B) Bioluminescence and (D) Fold gain in bioluminescence *vs.* 0 nM GDH over time after 15-60 mins pre-incubation. For all assays, GDH (final concentration indicated), 4-S (8 nM final concentration) and 4-L (8 nM final concentration) were incubated for the indicated length of time, at 25°C, with agitation prior to addition of Nano-Glo substrate to a final dilution of 1:1000. Data are the mean of duplicates on the same plate and error bars indicate standard deviation from the mean.

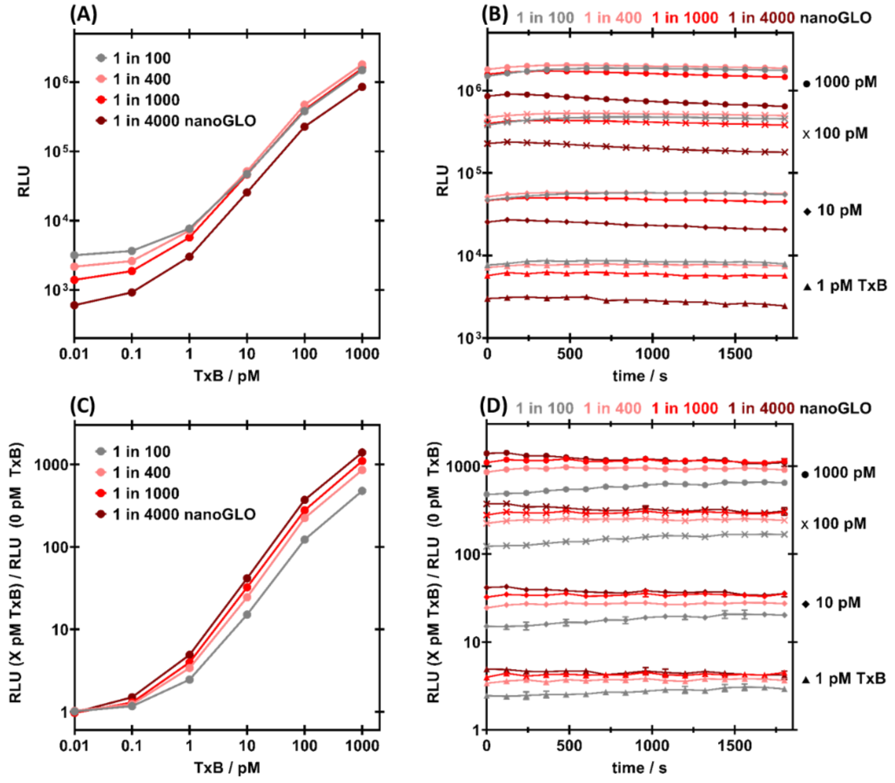

Figure S10. Establishing optimal Nano-Glo substrate concentration. (A) Bioluminescence and (C) Fold gain in bioluminescence dose response curve read immediately after substrate addition (B) Bioluminescence and (D) Fold gain in bioluminescence *vs.* 0 pM TxB over time. For all assays, TxB (final concentration indicated), S-E3 (0.5 nM final concentration) and L-45 (1 nM final concentration) were incubated for 30 mins, at 25°C, with agitation prior to addition of Nano-Glo substrate to a final dilution of 1:100 – 1:4000. Data are the mean of duplicates on the same plate and error bars indicate standard deviation from the mean.

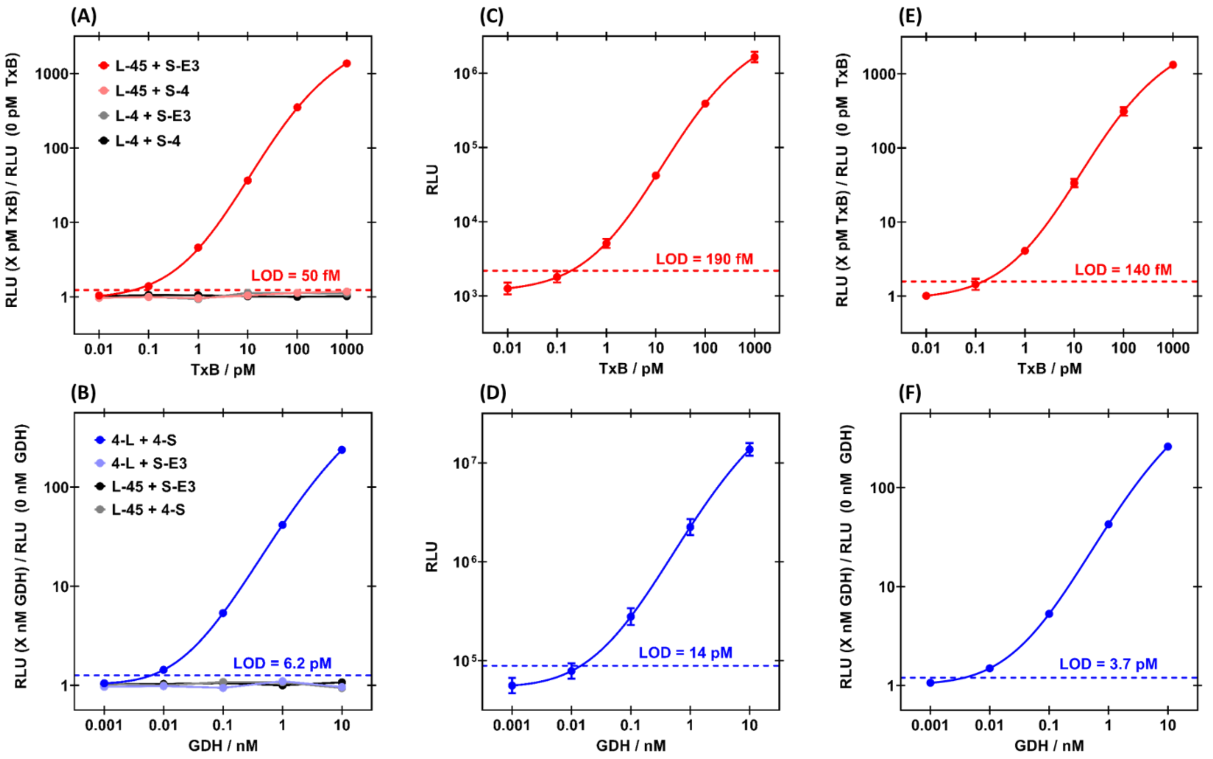

Figure S11. Optimised dose response curves used to calculate intra-assay (A, B) and inter-assay (C, D, E, F) LoD, accuracy (% recovery) and precision (% CV). (A) Fold gain response of TxB split luciferase assay with 1 nM LgBiT + 0.5 nM SmBiT. For L-45 + S-E3 (red) data are the mean of 6 replicates on the same plate and for control sensors (pink, grey, black) data are single measurements. (B) Fold gain response of GDH split luciferase assay with 8 nM LgBiT + 8 nM SmBiT. For 4-L + 4-S (blue) data are the mean of 6 replicates on the same plate and for control sensors (light blue, black, grey) data are single measurements. (C) Bioluminescent and (E) Fold gain response of TxB split luciferase assay with 0.5 nM S-E3 + 1 nM L-45. Data are the mean of 6 independent measurements. (D) Bioluminescent and (F) Fold gain response of GDH split luciferase assay with 8 nM 4-S + 8 nM 4-L. Data are the mean of 3 independent measurements. For all assays, analyte (final concentration indicated) and sensor proteins (final concentration indicated) were incubated for 30 mins, at 25°C, with agitation prior to addition of Nano-Glo substrate to a final dilution of 1:1000 and bioluminescence was read after 2 mins. Error bars indicate standard deviation from the mean, solid lines are 5PL regression fits and LoD indicated by dash line.

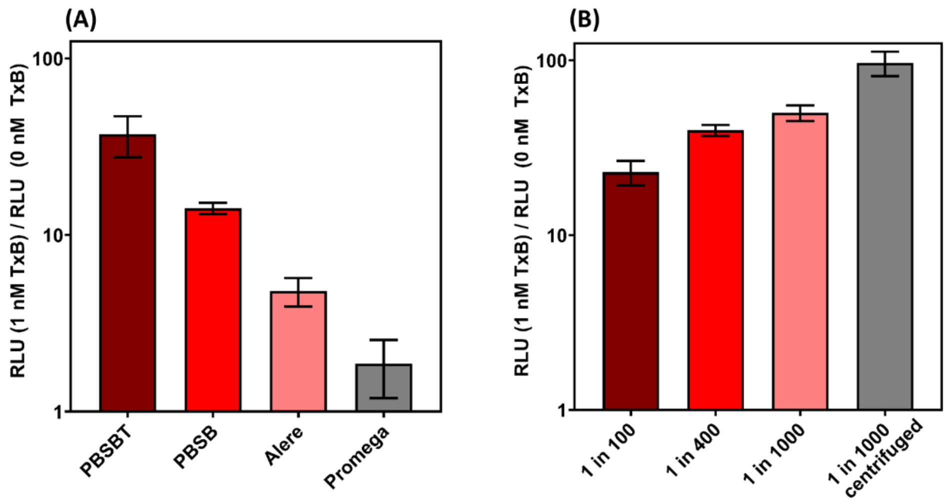

Figure S12. Fold gain response of the TxB split-luciferase assay in faecal sample matrix (A) Effect of buffer. Stool samples homogenised and assays performed in the stated buffer (PBSBT = PBS + 1 mg ml^{-1} BSA + 0.05 % Tween 20, PBSB = PBS + 1 mg ml^{-1} BSA, Alere = buffer used in *C. diff* Quik Chek complete test and Promega = buffer used in Nano-Glo kit). Final Nano-Glo concentration = 1:1000. (B) Effect of sample preparation and substrate concentration. Stool samples homogenised in PBSBT and particulates allowed to settle or pelleted by centrifugation, if stated. Final Nano-Glo concentration = 1:100 – 1:1000. For all assays, TxB (final concentration 1 nM), S-E3 (final concentration 0.5 nM) and L-45 (final concentration 1 nM) were incubated with *C. difficile* negative faecal sample (final concentration 0.66 % w/v) for 10 mins (A) or 30 mins (B), at 25°C, with agitation prior to addition of Nano-Glo. Bioluminescence was read after 2 mins. Data are the mean of duplicates on the same plate and error bars indicate standard deviation from the mean.

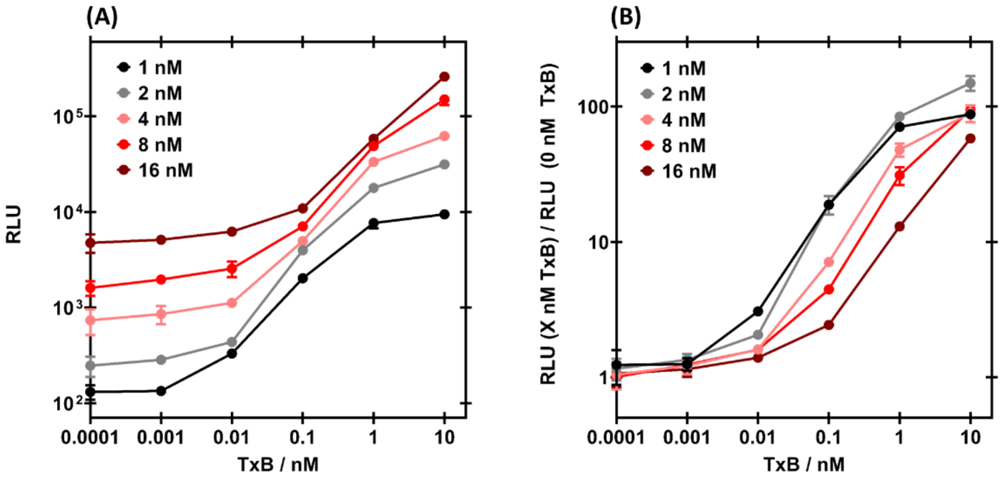

Figure S13. Effect of sensor protein concentrations on TxB dose response in faecal sample matrix. (A) Bioluminescent and (B) Fold gain response of 1 – 16 nM each of S-E3 + L-45. TxB (final concentration indicated), S-E3 (final concentration indicated) and L-45 (final concentration indicated) were incubated with *C. difficile* negative faecal sample (final concentration 0.66% w/v) for 30 mins, at 25°C, with agitation prior to addition of Nano-Glo substrate to a final concentration of 1:1000 and bioluminescence was read after 2 mins. Data are the mean of duplicates on the same plate and error bars indicate standard deviation from the mean.

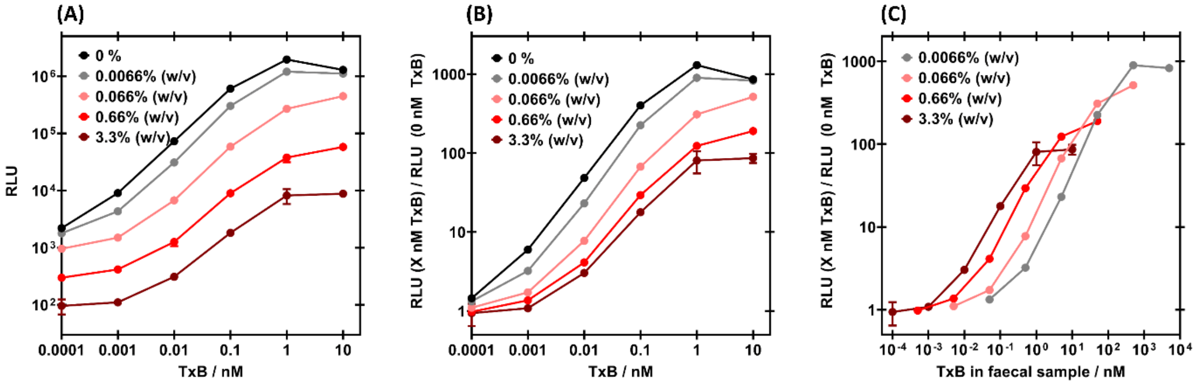

Figure S14. Effect of % faecal sample matrix on the TxB dose response (A) Bioluminescent and (B) Fold gain dose response to the final concentration of TxB in the assay with a 0 – 3.3% (w/v) faeces matrix (C) Fold gain dose response to the concentration of TxB in a 3.3% (w/v) faeces sample diluted in the assay to 0 – 0.66% (w/v) %. TxB, S-E3 (final concentration 0.5 nM) and L-45 (final concentration 1 nM) were incubated with *C. difficile* negative faecal sample (final concentration 3.33 - 0% (w/v)) for 30 mins, at 25°C, with agitation prior to addition of Nano-Glo to a final concentration of 1:1000 and bioluminescence was read after 2 mins. Data are the mean of duplicates on the same plate and error bars indicate standard deviation from the mean.

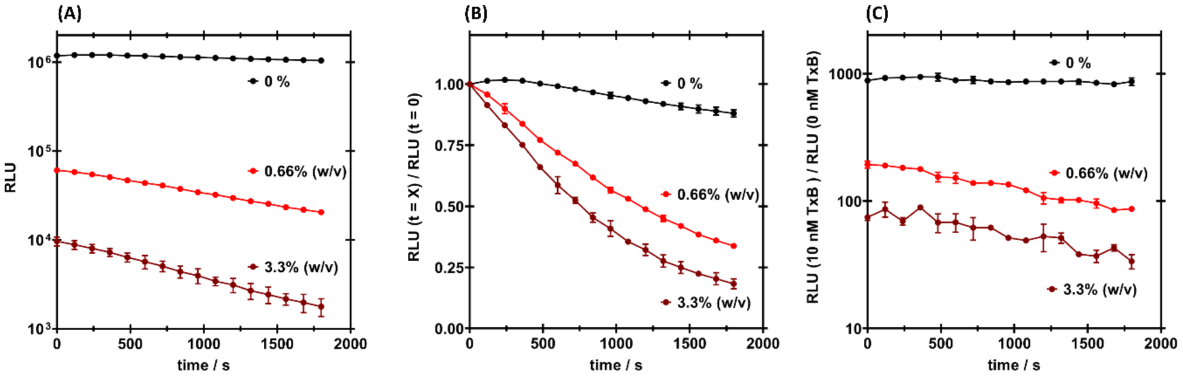

Figure S15. Effect of % faecal sample matrix on the TxB split-luciferase assay over time. (A) Bioluminescence (B) Fraction of initial bioluminescence and (C) Fold gain in bioluminescence *vs.* 0 nM TxB. TxB (10 nM final concentration), S-E3 (final concentration 0.5 nM) and L-45 (final concentration 1 nM) were incubated with *C. difficile* negative faecal sample (final concentration 3.33 - 0 % w/v) for 30 mins, at 25°C, with agitation prior to addition of Nano-Glo to a final concentration of 1:1000. Bioluminescence read immediately and then every 2 mins for 30 mins. Data are the mean of duplicates on the same plate and error bars indicate standard deviation from the mean.

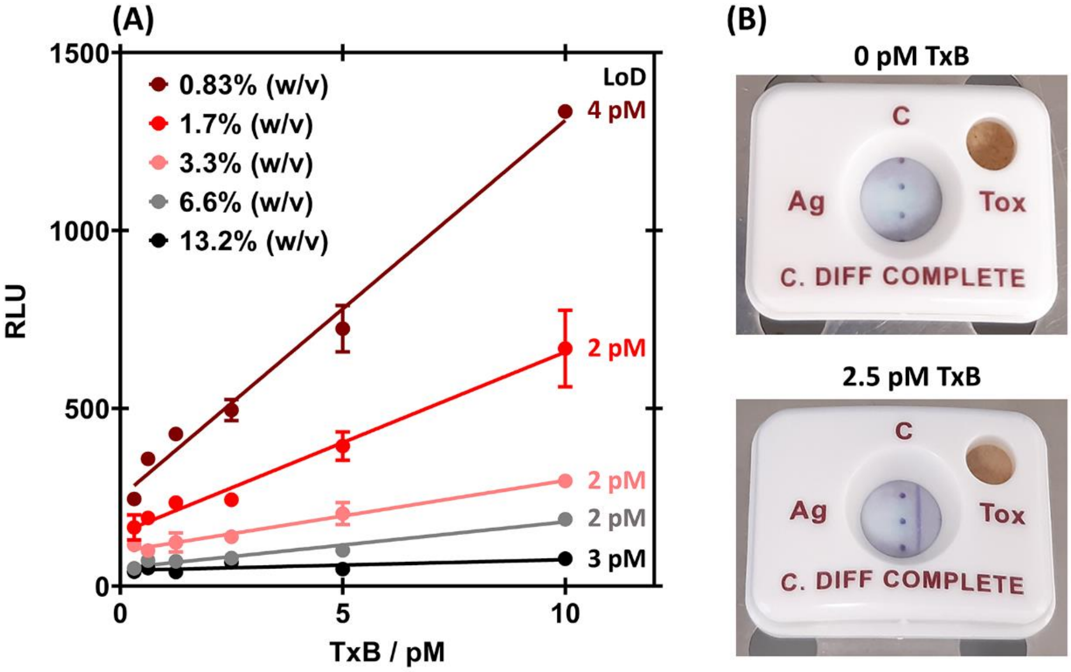

Figure S16. Measurement of the limit of detection (LoD) of the TxB split-luciferase assay *vs. C. diff* Quik Chek complete test (A) Bioluminescent response in a 0.83 – 13.2 % (w/v) faeces matrix with TxB at a final concentration of 0.3215 – 10 pM in the assay. TxB, S-E3 (final concentration 0.5 nM) and L-45 (1 nM final concentration) were incubated with *C. difficile* negative faecal sample (final concentration indicated) for 30 mins, at 25°C, with agitation prior to addition of Nano-Glo to a final concentration of 1:1000 and bioluminescence was read after 2 mins. Data are the mean of duplicates on the same plate and error bars indicate standard deviation from the mean. Data were fit to a simple linear regression (solid line) and LoD was calculated by LoD = $mean_{blank}$ + $1.645(SD_{blank}) + 1.645(SD_{2.5\ pM\ TxB})$ and corrected for concentration/dilution effects with respect to 3.3 % w/v faeces (e.g., the LoD at 6.6% (w/v) is 4 pM, but as faecal sample is twice as concentrated compared to 3.3% w/v, the value given is 2 pM). Optimal dilution for sensitivity centres around 3.3% w/v. (B) *C. diff* Quik Chek complete test performed to manufacturer's instructions on a *C. difficile* negative faecal sample (3.33 % w/v faeces) spiked with 0 or 2.5 pM TxB (approximately the LoD of the split-luciferase assay).

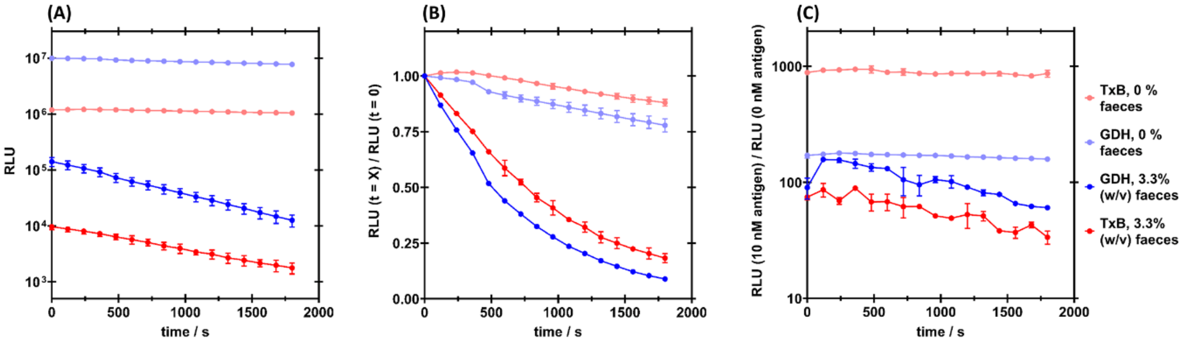

Figure S17. Effect of faeces on the TxB and GDH split-luciferase assays over time. (A) Bioluminescence (B) Fraction of initial bioluminescence and (C) Fold gain in bioluminescence *vs.* 0 nM analyte. Analyte (10 nM TxB or GDH, final concentration) and sensor proteins (0.5 nM S-E3 + 1 nM L-45, or 8 nM 4-S + 8nM 4-L, final concentrations) were incubated with *C. difficile* negative faecal sample (final concentration 3.33 or 0 % w/v) for 30 mins, at 25°C, with agitation prior to addition of Nano-Glo to a final concentration of 1:1000. Bioluminescence read immediately and then every 2 mins for 30 mins. Data are the mean of duplicates on the same plate and error bars indicate standard deviation from the mean.

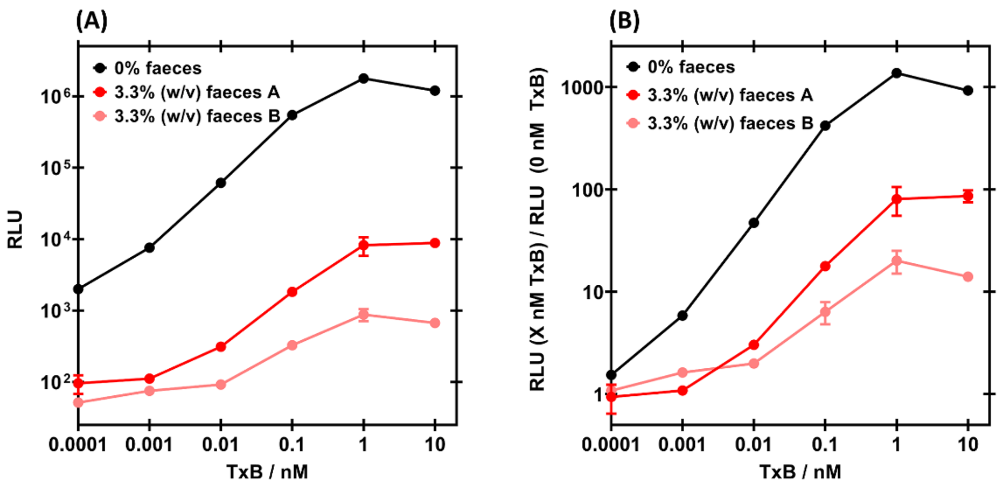

Figure S18. Effect of faecal samples from two different patients on the TxB split-luciferase assay (A) Bioluminescent and (B) Fold gain dose response curves with 0 % faeces (black), 3.33 % (w/v) patient A faeces and 3.33 % (w/v) patient B faeces. TxB, S-E3 (final concentration 0.5 nM) and L-45 (1 nM final concentration) were incubated with *C. difficile* negative faecal sample (final concentration indicated) for 30 mins, at 25°C, with agitation prior to addition of Nano-Glo to a final concentration of 1:1000 and bioluminescence was read after 2 mins. Data are the mean of duplicates on the same plate and error bars indicate standard deviation from the mean.

Table S1. Sensitivity (LoD), Accuracy (% recovery) and Precision (% CV) of TxB and GDH assays

		Intra-assay		Inter-assay	
		RLU	Fold gain	RLU	Fold Gain
TxB	Sensitivity (LoD)	44 fM	50 fM	190 fM	140 fM
	Accuracy (% recovery)	0.1–1000 pM (88–105 %)	1–1000 pM (97–105 %)	1–100 pM (99–101 %)	1–1000 pM (99–104 %)
	Precision (% CV)	0.1–1000 pM (3–25 %)*	1–1000 pM (4–7 %)	1–100 pM (11–20 %)	1–1000 pM (12–25 %)*
GDH	Sensitivity (LoD)	4.5 pM	6.2 pM	14 pM	3.7 pM
	Accuracy (% recovery)	0.01–10 nM (100 %)	0.01–10 nM (100-101 %)	0.1–10 nM (101–102 %)	0.01–10 nM (100 %)
	Precision (% CV)	0.01–10 nM (1–10 %)	0.01–10 nM (6–22 %)*	0.1–10 nM (19–24 %)*	0.01–10 nM (3–15 %)

Calculated from data in Figure 4 and S11. *%CV precision metrics >20% only at limit of quantification

DNA and Protein Sequences

Affimer 18

DNA

ATGGCTAGCAACTCCCTGGAAATCGAAGAACTGGCTCGTTTCGCTGTTGACGAACACAACAAAAAAGAAAACGCTCTGCT
GGAATTCGTTCGTGTTGTTAAAGCGAAAGAACAGGAAGAAACTAACGTTTACGGTAAAGACACCATGTACTACCTGACCC
TGGAAGCTAAAGACGGTGGTAAAAAGAAACTGTACGAAGCGAAAGTTTGGGTTAAGAGATTCAACAGATGGCCAAGTAAC
CTGAACTTCAAAGAACTGCAGGAGTTCAAACCGGTTGGTGACGCTGCGGCCGCGCATCACCATCATCACCACCATCAT

Protein

MASNSLEIEELARFAVDEHNKKENALLEFVRVVKAKEQEETNVYGKDTMYYLTLEAKDGGKKKLYEAKVWVKRFNRWPSN
LNFKELQEFKPVGDAAAAHHHHHHHH

Affimer 45

DNA

ATGGCTAGCAACTCCCTGGAAATCGAAGAACTGGCTCGTTTCGCTGTTGACGAACACAACAAAAAAGAAAACGCTCTGCT
GGAATTCGTTCGTGTTGTTAAAGCGAAAGAACAGGAACAGCGTCATAAACATGCTACTTTCACCATGTACTACCTGACCC
TGGAAGCTAAAGACGGTGGTAAAAAGAAACTGTACGAAGCGAAAGTTTGGGTTAAGAACAACAACAGAGCAATGTTCATG
ACCAACTTCAAAGAACTGCAGGAGTTCAAACCGGTTGGTGACGCTGCGGCCGCGCATCACCATCATCACCACCATCAT

Protein

MASNSLEIEELARFAVDEHNKKENALLEFVRVVKAKEQEQRHKHATFTMYYLTLEAKDGGKKKLYEAKVWVKNNNRAMFM
TNFKELQEFKPVGDAAAAHHHHHHHH

Affimer 4

DNA

ATGGCTAGCAACTCCCTGGAAATCGAAGAACTGGCTCGTTTCGCTGTTGACGAACACAACAAAAAAGAAAACGCTCTGCT
GGAATTCGTTCGTGTTGTTAAAGCGAAAGAACAGCATGTTACTCAGTTCGACTCTTTCGCTACCATGTACTACCTGACCC
TGGAAGCTAAAGACGGTGGTAAAAAGAAACTGTACGAAGCGAAAGTTTGGGTTAAGAGTAACCATGGCTTCTTCCAGCAG
GAAAACTTCAAAGAACTGCAGGAGTTCAAACCGGTTGGTGACGCTGCGGCCGCGCATCACCATCATCACCACCATCAT

Protein

MASNSLEIEELARFAVDEHNKKENALLEFVRVVKAKEQHVTQFDSFATMYYLTLEAKDGGKKKLYEAKVWVKSNHGFFQQ
ENFKELQEFKPVGDAAAAHHHHHHHH

Nanobody E3

DNA

CAGGTGCAACTGGTTGAAAGCGGTGGCGGTCTGGTGCAAACCGGCGGTAGCCTGCGTCTGAGCTGCGCGAGCAGCGGTAG
CATTGCGGGTTTCGAAACCGTGACCTGGAGCCGTCAGGCGCCGGGCAAGAGCCTGCAATGGGTTGCGAGCATGACCAAAA
CCAACAACGAAATTTACAGCGACAGCGTTAAGGGTCGTTTCATCATTAGCCGTGATAACGCGAAAAACACCGTGTACCTG
CAGATGAACAGCCTGAAGCCGGAGGACACCGGCGTTTATTTTTGCAAAGGTCCGGAACTGCGTGGCCAGGGTATTCAGGT
GACCGTTAGCAGC

Protein

QVQLVESGGGLVQTGGSLRLSCASSGSIAGFETVTWSRQAPGKSLQWVASMTKTNNEIYSDSVKGRFIISRDNAKNTVYL
QMNSLKPEDTGVYFCKGPELRGQGIQVTVSS

Nanobody 7F

DNA

CAGGTGCAACTGGTTGAGAGCGGTGGCGGTCTGGTGGAAGCGGGCGGTAGCCTGCGTCTGAGCTGCGTGGTTACCGGCAG
CAGCTTTAGCACCAGCACGATGGCGTGGTACCGTCAGCCGCCGGGCAAGCAACGTGAATGGGTGGCGAGCTTCACCAGCG

GCGGTGCGATCAAGTACACCGACAGCGTTAAAGGTCGTTTTACCATGAGCCGTGATAACGCGAAGAAAATGACCTATCTG
CAGATGGAGAACCTGAAACCGGAAGACACCGCGGTGTACTATTGCGCGCTGCATAACGCGGTTAGCGGTAGCAGCTGGGG
TCGTGGTACCCAAGTGACCGTTAGCAGC

Protein

QVQLVESGGGLVEAGGSLRLSCVVTGSSFSTSTMAWYRQPPGKQREWVASFTSGGAIKYTDSVKGRFTMSRDNAKKMTYL
QMENLKPEDTAVYYCALHNAVSGSSWGRGTQVTVSS

LgBiT

DNA

GTTTTTACCCTGGAAGATTTCGTGGGCGACTGGGAACAGACCGCGGCGTACAACCTGGACCAAGTGCTGGAACAA
GGTGGCGTGAGCAGCCTGCTGCAGAACCTGGCGGTGAGCGTTACCCCGATCCAACGTATTGTTCGTAGCGGCGAG
AACGCGCTGAAGATCGACATTCACGTGATCATTCCGTACGAAGGCCTGAGCGCGGATCAGATGGCGCAAATCGAG
GAAGTGTTCAAGGTGGTTTACCCGGTTGACGATCACCACTTTAAAGTGATCCTGCCGTATGGTACCCTGGTGATT
GACGGCGTTACCCCGAACATGCTGAACTACTTCGGTCGTCCGTATGAGGGCATCGCGGTTTTTGATGGTAAGAAA
ATTACCGTGACCGGTACCCTGTGGAACGGCAACAAAATTATTGATGAGCGCCTGATTACCCCGGACGGCAGCATG
CTGTTTCGTGTGACCATTAATAGC

Protein

VFTLEDFVGDWEQTAAYNLDQVLEQGGVSSLLQNLAVSVTPIQRIVRSGENALKIDIHVIIPYEGLSADQMAQIEEVFKV
VYPVDDHHFKVILPYGTLVIDGVTPNMLNYFGRPYEGIAVFDGKKITVTGTLWNGNKIIDERLITPDGSMLFRVTINS

Sensors

All sensors have SmBit (101) or LgBiT at one terminus and an Affimer or nanobody at the other terminus, with a GSG_7 linker in between and a C-terminal Histag. The sequences of the optimal sensors for TxB (L-45, S-E3) and GDH (4-L, 4-S) are shown in full below.

Key to sensor construct components:

Blue highlight = LgBiT, red highlight = SmBiT (101), green highlight = GSG_7, yellow highlight = Affimer (Aff), grey highlight = nanobody (NB) and pink highlight = Histag.

TxB sensor L-45

(LgBit-GSG_7-Aff45-Histag)

DNA

ATGGCGGCTAGCGTTTTTACCCTGGAAGATTTCGTGGGCGACTGGGAACAGACCGCGGCGTACAACCTGGACCAAGTGCT
GGAACAAGGTGGCGTGAGCAGCCTGCTGCAGAACCTGGCGGTGAGCGTTACCCCGATCCAACGTATTGTTCGTAGCGGCG
AGAACGCGCTGAAGATCGACATTCACGTGATCATTCCGTACGAAGGCCTGAGCGCGGATCAGATGGCGCAAATCGAGGAA
GTGTTCAAGGTGGTTTACCCGGTTGACGATCACCACTTTAAAGTGATCCTGCCGTATGGTACCCTGGTGATTGACGGCGT
TACCCCGAACATGCTGAACTACTTCGGTCGTCCGTATGAGGGCATCGCGGTTTTTGATGGTAAGAAAATTACCGTGACCG
GTACCCTGTGGAACGGCAACAAAATTATTGATGAGCGCCTGATTACCCCGGACGGCAGCATGCTGTTTCGTGTGACCATT
AATAGCGCGGCCGCTGGGTCCGGCGGTTCAGGCGGCTCTGGTGGCTCCGGTGGGTCAGGTGGTTCTGGCGGGTCTGGCAC
TAGTGCAAACTCCCTGGAAATCGAAGAACTGGCTCGTTTCGCTGTTGACGAACACAACAAAAAAGAAAACGCTCTGCTGG
AATTCGTTCGTGTTGTTAAAGCGAAAGAACAGGAACAGCGTCATAAACATGCTACTTTCACCATGTACTACCTGACCCTG
GAAGCTAAAGACGGTGGTAAAAAGAAACTGTACGAAGCGAAAGTTTGGGTTAAGAACAACAACAGAGCAATGTTCATGAC
CAACTTCAAAGAACTGCAGGAGTTCAAACCAGTAGTCGACCTCGAGCACCACCACCACCACCAC

Protein

MAASVFTLEDFVGDWEQTAAYNLDQVLEQGGVSSLLQNLAVSVTPIQRIVRSGENALKIDIHVIIPYEGLSADQMAQIEE
VFKVVYPVDDHHFKVILPYGTLVIDGVTPNMLNYFGRPYEGIAVFDGKKITVTGTLWNGNKIIDERLITPDGSMLFRVTI
NSAAAGSGGSGGSGGSGGSGGSGGSGTSANSLEIEELARFAVDEHNKKENALLEFVRVVKAKEQEQRHKHATFTMYYLTL
EAKDGGKKKLYEAKVWVKNNNRAMFMTNFKELQEFKPVVDLEHHHHHH

TxB sensor S-E3

(SmBiT101-GSG_7-NBE3-Histag)

DNA

ATGGCGGCTAGCGTAACAGGATATAGGCTATTTGAAAAAGAGAGCGCGGCCGCTGGGTCCGGCGGTTCAGGCGGCTCTGG
TGGCTCCGGTGGGTCAGGTGGTTCTGGCGGGTCTGGCACTAGTCAGGTGCAACTGGTTGAAAGCGGTGGCGGTCTGGTGC
AAACCGGCGGTAGCCTGCGTCTGAGCTGCGCGAGCAGCGGTAGCATTGCGGGTTTCGAAACCGTGACCTGGAGCCGTCAG
GCGCCGGGCAAGAGCCTGCAATGGGTTGCGAGCATGACCAAAACCAACAACGAAATTTACAGCGACAGCGTTAAGGGTCG
TTTCATCATTAGCCGTGATAACGCGAAAAACACCGTGTACCTGCAGATGAACAGCCTGAAGCCGGAGGACACCGGCGTTT
ATTTTTGCAAAGGTCCGGAACTGCGTGGCCAGGGTATTCAGGTGACCGTTAGCAGCGTCGACCTCGAGCACCACCACCAC
CACCAC

Protein

MAASVTGYRLFEKESAAAGSGGSGGSGGSGGSGGSGGSGTSQVQLVESGGGLVQTGGSLRLSCASSGSIAGFETVTWSRQ
APGKSLQWVASMTKTNNEIYSDSVKGRFIISRDNAKNTVYLQMNSLKPEDTGVYFCKGPELRGQGIQVTVSSVDLEHHHH
HH

GDH sensor 4-L

(Aff4-GSG_7-LgBiT-Histag)

DNA

ATGGCGGCTAGCAACTCCCTGGAAATCGAAGAACTGGCTCGTTTCGCTGTTGACGAACACAACAAAAAAGAAAACGCTCT
GCTGGAATTCGTTCGTGTTGTTAAAGCGAAAGAACAGCATGTTACTCAGTTCGACTCTTTCGCTACCATGTACTACCTGA
CCCTGGAAGCTAAAGACGGTGGTAAAAAGAAACTGTACGAAGCGAAAGTTTGGGTTAAGAGTAACCATGGCTTCTTCCAG
CAGGAAAACTTCAAAGAACTGCAGGAGTTCAAACCAGTAGCGGCCGCTGGGTCCGGCGGTTCAGGCGGCTCTGGTGGCTC
CGGTGGGTCAGGTGGTTCTGGCGGGTCTGGCACTAGTGTTTTTACCCTGGAAGATTTCGTGGGCGACTGGGAACAGACCG
CGGCGTACAACCTGGACCAAGTGCTGGAACAAGGTGGCGTGAGCAGCCTGCTGCAGAACCTGGCGGTGAGCGTTACCCCG
ATCCAACGTATTGTTCGTAGCGGCGAGAACGCGCTGAAGATCGACATTCACGTGATCATTCCGTACGAAGGCCTGAGCGC
GGATCAGATGGCGCAAATCGAGGAAGTGTTCAAGGTGGTTTACCCGGTTGACGATCACCACTTTAAAGTGATCCTGCCGT
ATGGTACCCTGGTGATTGACGGCGTTACCCCGAACATGCTGAACTACTTCGGTCGTCCGTATGAGGGCATCGCGGTTTTT
GATGGTAAGAAAATTACCGTGACCGGTACCCTGTGGAACGGCAACAAAATTATTGATGAGCGCCTGATTACCCCGGACGG
CAGCATGCTGTTTCGTGTGACCATTAATAGCGTCGACCTCGAGCACCACCACCACCACCAC

Protein

MAASNSLEIEELARFAVDEHNKKENALLEFVRVVKAKEQHVTQFDSFATMYYLTLEAKDGGKKKLYEAKVWVKSNHGFFQ
QENFKELQEFKPVAAAGSGGSGGSGGSGGSGGSGGSGTSVFTLEDFVGDWEQTAAYNLDQVLEQGGVSSLLQNLAVSVTP
IQRIVRSGENALKIDIHVIIPYEGLSADQMAQIEEVFKVVYPVDDHHFKVILPYGTLVIDGVTPNMLNYFGRPYEGIAVF
DGKKITVTGTLWNGNKIIDERLITPDGSMLFRVTINSVDLEHHHHHH

GDH sensor 4-S

(Aff4-GSG_7-SmBiT101-Histag)

DNA

ATGGCGGCTAGCAACTCCCTGGAAATCGAAGAACTGGCTCGTTTCGCTGTTGACGAACACAACAAAAAAGAAAACGCTCT
GCTGGAATTCGTTCGTGTTGTTAAAGCGAAAGAACAGCATGTTACTCAGTTCGACTCTTTCGCTACCATGTACTACCTGA
CCCTGGAAGCTAAAGACGGTGGTAAAAAGAAACTGTACGAAGCGAAAGTTTGGGTTAAGAGTAACCATGGCTTCTTCCAG
CAGGAAAACTTCAAAGAACTGCAGGAGTTCAAACCAGTAGCGGCCGCTGGGTCCGGCGGTTCAGGCGGCTCTGGTGGCTC
CGGTGGGTCAGGTGGTTCTGGCGGGTCTGGCACTAGTGTAACAGGATATAGGCTATTTGAAAAAGAGAGCGTCGACCTCG
AGCACCACCACCACCACCAC

Protein

MAASNSLEIEELARFAVDEHNKKENALLEFVRVVKAKEQHVTQFDSFATMYYLTLEAKDGGKKKLYEAKVWVKSNHGFFQ
QENFKELQEFKPVAAAGSGGSGGSGGSGGSGGSGGSGTSVTGYRLFEKESVDLEHHHHHH

Tables of Primers

Table S2. Primers used for Affimer amplification

Primer	Sequence
NheI-Aff-f	ATGCGCTAGCAACTCCCTGGAAATCGAAGAACTG
NotI-Aff-r	TAATGCGGCCGCTACTGGTTTGAACTCCTGCAGTTCTTTG
SpeI-Aff-f	AACGACTAGTGCAAACTCCCTGGAAATCGAAGAACTG
SalI-Aff-r	TAATGTCGACTACTGGTTTGAACTCCTGCAGTTCTTTG

Table S3. Primers used for nanobody amplification

Primer	Sequence
NheI-7F-f	TAATGCTAGCCAGGTGCAACTGGTTGAGAG
NotI-7F-r	TTATGCGGCCGCGCTGCTAACGGTCACTTGG
SpeI-7F-f	TAATACTAGTCAGGTGCAACTGGTTGAGAG
SalI-7F-r	TAATGTCGACGCTGCTAACGGTCACTTGG
NheI-E3-f	TAATGCTAGCCAGGTGCAACTGGTTGAAAGC
NotI-E3-r	TTATGCGGCCGCGCTGCTAACGGTCACCTG
SpeI-E3-f	TAATACTAGTCAGGTGCAACTGGTTGAAAGC
SalI-E3-r	TAATGTCGACGCTGCTAACGGTCACCTG

Table S4. Primers used for LgBiT amplification

Primer	Sequence
LgBiT-NheI-f	TAATGCTAGCGTTTTTACCCTGGAAGATTTCG
LgBiT-NotI-r	TTATGCGGCCGCGCTATTAATGGTCACACGAAAC
LgBiT-SpeI-f	TAAAACTAGTGTTTTTACCCTGGAAGATTTCG
LgBiT-SalI-r	TAATGTCGACGCTATTAATGGTCACACGAAAC

Table S5. Primers used to generate SmBiT (101)

Primer	Sequence
SmBiT101-NheI-f	TAATGCTAGCGTAACAGGATATAGGCTATTTGAAAAAGAGAGC
SmBiT101-NotI-r	TTATGCGGCCGCGCTCTCTTTTTCAAATAGCCTATATCCTGTTAC
SmBiT101-SpeI-f	TAAAACTAGTGTAACAGGATATAGGCTATTTGAAAAAGAGAGC
SmBiT101-SalI-r	TAATGTCGACGCTCTCTTTTTCAAATAGCCTATATCCTGTTAC

Table S6. Primers used to generate GSG_7 linker

Primer	Sequence
NotI-GSG_7-f	TTATGCGGCCGCTGGGTCCGGCGGTTCAGGCGGCTCTGGTGGCTCCGGTGGGTCAGGT
SpeI-GSG_7-r	TAAAACTAGTGCCAGACCCGCCAGAACCACCTGACCCACCGGAGCCACCAGAGCC

References

1. Tiede, C.; Tang, A. A.; Deacon, S. E.; Mandal, U.; Nettleship, J. E.; Owen, R. L.; George, S. E.; Harrison, D. J.; Owens, R. J.; Tomlinson, D. C., *Protein Eng. Des. Sel.* **2014,** *27*, 145-155.
2. Ni, Y.; Rosier, B. J.; van Aalen, E. A.; Hanckmann, E. T.; Biewenga, L.; Pistikou, A.-M. M.; Timmermans, B.; Vu, C.; Roos, S.; Arts, R.; Li, W.; de Greef, T. F.; van Borren, M. M.; van Kuppeveld, F. J.; Bosch, H.-J.; Merkx, M., *Nat. Commun.* **2021,** *12*, 4586.
3. Dixon, A. S.; Schwinn, M. K.; Hall, M. P.; Zimmerman, K.; Otto, P.; Lubben, T. H.; Butler, B. L.; Binkowski, B. F.; Machleidt, T.; Kirkland, T. A., *ACS Chem. Biol.* **2016,** *11*, 400-408.
